# ECM microenvironment unlocks brown adipogenic potential of adult human bone marrow-derived MSCs

**DOI:** 10.1038/srep21173

**Published:** 2016-02-17

**Authors:** Michelle H. Lee, Anna G. Goralczyk, Rókus Kriszt, Xiu Min Ang, Cedric Badowski, Ying Li, Scott A. Summers, Sue-Anne Toh, M. Shabeer Yassin, Asim Shabbir, Allan Sheppard, Michael Raghunath

**Affiliations:** 1 Department of Biomedical Engineering, National University of Singapore, 117575, Singapore; 2 NUS Graduate School for Integrative Sciences and Engineering (NGS), National University of Singapore, 117456, Singapore; 3 NUS Tissue Engineering Program, Life Science Institute, National University of Singapore, 117510, Singapore; 4 Institute of Medical Biology, A*STAR, 138648, Singapore; 5 Program in Cardiovascular and Metabolic Diseases, Duke-NUS Medical Graduate School, 169857, Singapore; 6 Translational Metabolic Health Laboratory, Baker IDI Heart and Diabetes Institute, Melbourne VIC 3004, Australia; 7 Department of Medicine, National University Health System, 119228, Singapore; 8 Department of Medicine, Yong Loo Lin School of Medicine, National University of Singapore, 117599, Singapore; 9 Department of Surgery, National University Hospital, 119074, Singapore; 10 Liggins Institute, University of Auckland, Auckland 1142 New Zealand; 11 Department of Biochemistry, Yong Loo Lin School of Medicine, National University of Singapore, 117599, Singapore

## Abstract

Key to realizing the diagnostic and therapeutic potential of human brown/brite adipocytes is the identification of a renewable, easily accessible and safe tissue source of progenitor cells, and an efficacious *in vitro* differentiation protocol. We show that macromolecular crowding (MMC) facilitates brown adipocyte differentiation in adult human bone marrow mesenchymal stem cells (bmMSCs), as evidenced by substantially upregulating uncoupling protein 1 (UCP1) and uncoupled respiration. Moreover, MMC also induced ‘browning’ in bmMSC-derived white adipocytes. Mechanistically, MMC creates a 3D extracellular matrix architecture enshrouding maturing adipocytes in a collagen IV cocoon that is engaged by paxillin-positive focal adhesions also at the apical side of cells, without contact to the stiff support structure. This leads to an enhanced matrix-cell signaling, reflected by increased phosphorylation of ATF2, a key transcription factor in UCP1 regulation. Thus, tuning the dimensionality of the microenvironment *in vitro* can unlock a strong brown potential dormant in bone marrow.

Brown adipocytes (BA) facilitate non-shivering thermogenesis during cold exposure or diet-induced thermogenesis via UCP1 which resides in the mitochondrial membrane of these cells. Extensive work in mice suggests the existence of at least two populations of BA. “Classical” BA reside in the interscapular region, while ‘beige/brite’ adipocytes^1^ are embedded within white adipose tissue (WAT) deposits and can become thermogenic, expressing UCP1 under certain conditions such as β-adrenergic stimulation or chronic PPARγ agonist exposure^2^.

In humans, substantial amounts of classical BA are similarly present in the interscapular region, but only during infancy^3^. In adults, 18F-fluorodeoxyglucose studies and imaging-directed biopsies locate thermogenic UCP1-positive adipose tissue to the supraclavicular and neck region^4^,^5^,^6^, but it appears to comprise mainly brite adipocytes^7^,^8^ or a mixture of brite and classical BA^9^,^10^. Epidemiological studies suggest a correlation of increased activity of these brown/brite fat depots with smaller body mass^11^. In conjunction with previous estimates that in humans as little as 50 g of maximally stimulated BAT could account for 20% of daily energy expenditure^12^, an enormous latent therapeutic potential is currently ascribed to endogenous brown/brite adipocytes for tackling obesity and co-morbidities such as type 2 diabetes, and metabolic syndrome^13^. From a cell therapeutic perspective this would require autologous progenitor cells that could be converted into BA. From a pharmaceutical perspective, a sufficient amount of human BA would be required to directly screen for thermogenic or browning pharmaceutical and nutraceutical components^14^. Two issues arise here. Firstly, a renewable source of BA progenitors is needed that can be accessed with ease. The currently described locations of UCP1-expressing adipocytes in humans require image-guided tissue biopsies close to large blood vessels (supraclavicular, lateral neck), open chest surgery (mediastinum) or parental consent (prepubic fat pads in infants)^15^,^16^,^17^. Obviously, these anatomical locations preclude straightforward and repeatable access to BA progenitors. Secondly, a robust protocol needs to be in place that facilitates the differentiation of human progenitors into BA efficaciously without genetic manipulation. This is not trivial, as current models of brown/brite cell differentiation and functionality (and related nomenclature discussions) are almost exclusively based on work in mice and murine cell lines^18^. Attempts have been made to employ substantial reprogramming of starting material, including iPS generation^19^,^20^. We address here both issues demonstrating that bone marrow-derived mesenchymal stromal cells and the stromal vascular fraction of subcutaneous (SC) tissue of human adults contains progenitor cells with dormant thermogenic potential that can be unleashed with a specific differentiation protocol that makes use of a novel principle in tissue engineering, macromolecular crowding.

## Results

### Macromolecular crowding (MMC) enhances adipogenic differentiation towards a brown adipocyte phenotype in adult human bone marrow mesenchymal stem cells

**Figure 1:**
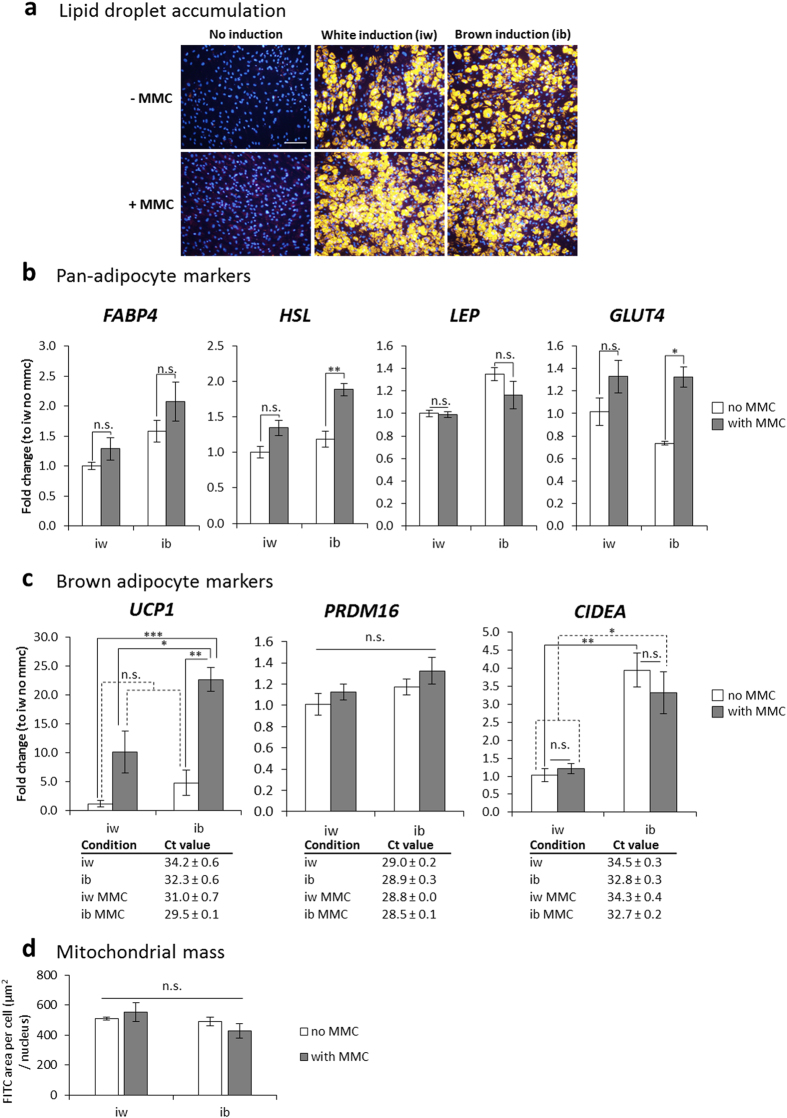
Differentiation of bmMSCs into adipocytes under macromolecular crowding (MMC) upregulates *UCP1* expression. (**a**) Fluorescence images of Nile Red-stained lipid droplets (yellow) and DAPI-stained nuclei (blue) at 10X magnification. Scale bar: 200 μm. (**b**) qPCR analysis of pan-adipocyte genes: *FABP4* = fatty acid binding protein 4, *HSL* = hormone sensitive lipase, *LEP* = leptin, *GLUT4* = glucose transporter type 4. (**c**) qPCR analysis of BAT selective genes: *UCP1* = uncoupling protein 1, *PRDM16* = PRD1-BF1-RIZ1 homologous domain containing 16, *CIDEA* = cell death-inducing DNA fragmentation factor, alpha subunit-like effector a. (**d**) Quantification of mitochondrial mass using mitotracker Green normalized to cell number. Data is expressed as mean ± SEM. n.s. =not significant; *p < 0.05; **p < 0.01; ***p < 0.001. N = 3.

Adult human bone marrow mesenchymal stem cells (bmMSCs) were subjected to a standard white (iw) protocol (four factors: IBMX, indomethacin, dexamethasone and insulin^21^,^22^) or a brown (ib) adipogenic induction protocol for 3 weeks. The ib induction was adapted from work in murine cells with the addition of factors known to promote brown adipocyte differentiation, namely T3^23^, PPARγ agonist rosiglitazone^24^ and bone morphogenetic protein BMP7^25^. In addition, these combinations were tested in the presence of MMC using a mixture of Ficoll70 and 400 with a combined fractional volume occupancy of 18% (v/v)^26^ and as recently applied in WAT differentiation^27^. While MMC alone did not induce adipogenic differentiation (Fig. 1a), both white (iw) and brown (ib) adipogenic induction ±MMC induced substantial lipid droplet accumulation (Fig. 1a) and expression of pan-adipocyte markers (Fig. 1b). As expected, the iw protocol did not induce any *UCP1* expression in the differentiated adipocytes (Fig. 1c). The ib protocol also failed to significantly upregulate *UCP1* expression in bmMSCs. However, in the presence of MMC (ib MMC) an over 20-fold *UCP1* expression occurred, compared to the iw protocol, and 5-fold compared to ib protocol alone (Fig. 1c). Surprisingly, MMC also upregulated *UCP1* expression with the iw protocol (iw MMC) by 10-fold, in the absence of the browning factors (Fig. 1c). Expression levels of other brown-related genes such as *PRDM16* and *CIDEA* were not significantly different between groups (Fig. 1c). Mitochondrial mass also did not differ between groups (Fig. 1d).

### MMC-generated brown adipocytes (BA) derived from adult human bone marrow MSCs are functional

**Figure 2:**
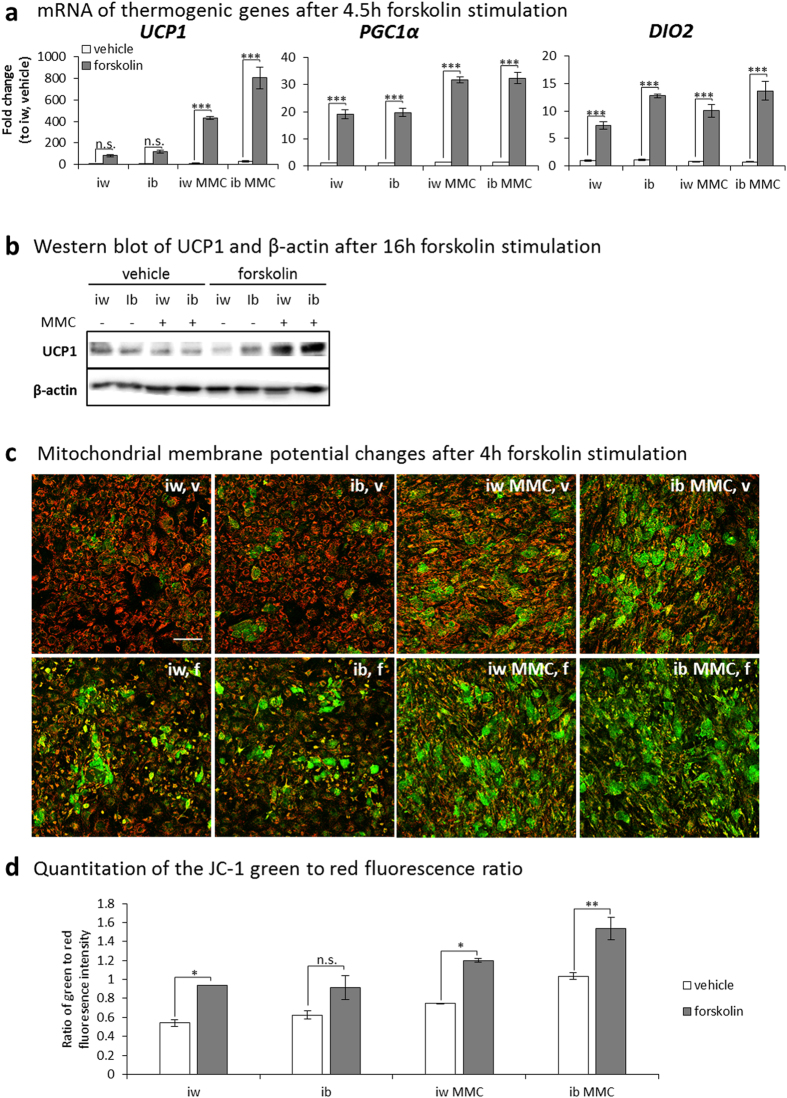
UCP1-expressing bmMSC-derived adipocytes show upregulation of thermogenic genes, UCP1 protein and membrane depolarization, upon forskolin stimulation. bmMSC-derived adipocytes were subjected to a forskolin (10 μM) stimulus, and thermogenic gene expression, UCP1 protein and mitochondrial membrane depolarization were assessed. (**a**) qPCR to determine mRNA expression of thermogenic genes after a 4.5 h forskolin stimulus. *UCP1* = uncoupling protein 1; *PGC1α* = PPARγ co-activator 1α; *DIO2* = deiodinase, iodothyronine, type II. Data is expressed as mean ± SEM. n.s. =not significant; *p < 0.05; **p < 0.01; ***p < 0.001. N = 3. (**b**) Western blotting of UCP1 and β-actin after a 16 h forskolin stimulus. (**c**) JC-1 staining of bmMSC-derived adipocytes after a 4 h forskolin stimulus (f) compared to vehicle (v) controls. Scale bar: 200 μm. (**d**) Quantitation of the JC-1 green to red fluorescence ratio of the images. Data is expressed as mean ± SEM. n.s. =not significant; *p < 0.05; **p < 0.01; ***p < 0.001. Measurements of two images per condition were taken (technical duplicate) to obtain the data in (**d**).

**Figure 3:**
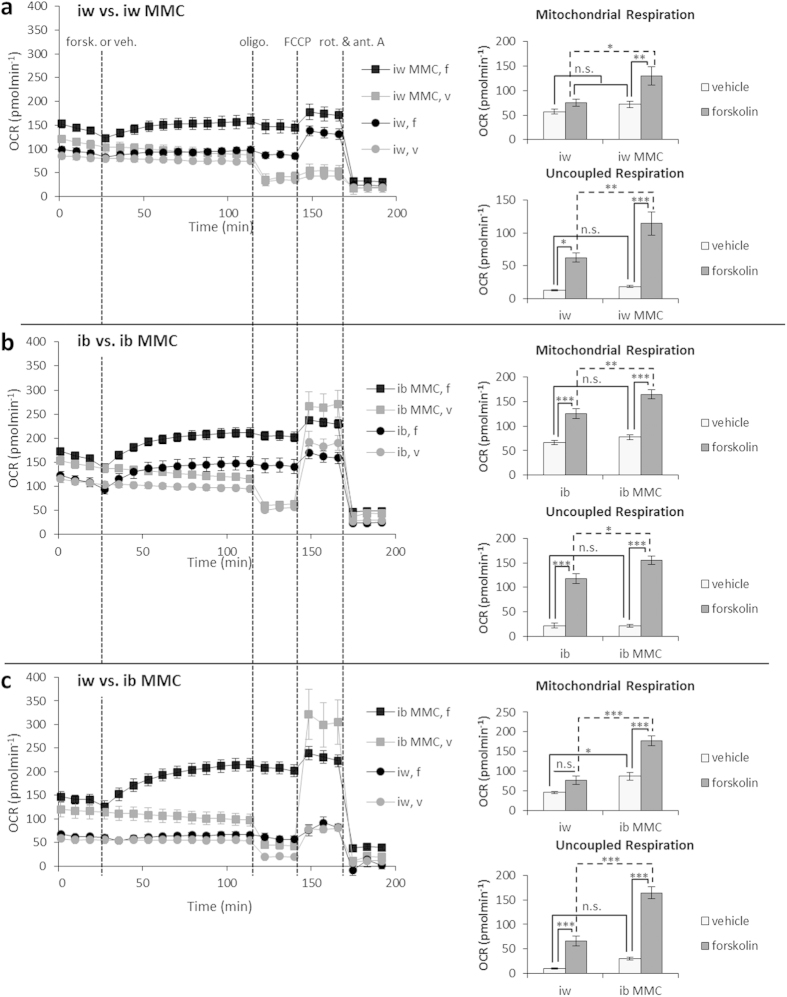
UCP1-expressing bmMSC-derived adipocytes show increased mitochondrial and uncoupled respiration upon forskolin stimulation. Oxidative respiratory capacity (OCR) was measured in bmMSC-derived adipocytes ± MMC to assess the extent of increase in mitochondrial and uncoupled respiration in response to a forskolin (10 μM) stimulus. Respiratory profiles of (**a**) iw vs. iw MMC, (**b**) ib vs. ib MMC, and (**c**) iw vs. ib MMC. Data is expressed as mean ± SEM. n.s. =not significant; *p < 0.05; **p < 0.01; ***p < 0.001. N = 5.

To evaluate the functionality of the MMC-generated BA, we first investigated their lipolytic response, as both white and brown adipocytes perform lipolysis upon a β-adrenergic stimulus^28^. Emulating a downstream signaling event, we applied a 16 h forskolin stimulus; both iw- and ib- generated adipocytes ±MMC responded by emptying their lipid stores by 50%, a clear sign of lipolysis (Fig. S1). After a 4.5 h forskolin stimulus all MMC-generated adipocytes showed a greater upregulation of thermogenic genes *PGC1α* and *DIO2* compared to induction with cocktail alone. *UCP1* upregulation was only significant with iw MMC (430-fold) and ib MMC (800-fold) compared to iw unstimulated (Fig. 2a). These adipocytes also responded to norepinephrine (Fig. S2). Concurrently, UCP1 protein was also upregulated to a greater extent in the MMC conditions after a 16 h forskolin stimulus (Figs 2b and S3). MMC-generated adipocytes (iw MMC and ib MMC) showed a greater extent of mitochondrial membrane depolarization (JC-1 staining) which predicted increased UCP1 activity^29^ (Fig. 2c, lower panel; Figs 2d and S4). To investigate the extent of increase in mitochondrial and uncoupled respiration during thermogenesis, the adipocytes were stimulated with forskolin and the oxygen consumption rate (OCR) was measured real time, after which metabolic inhibitors were added in sequence to ascertain mitochondrial and uncoupled respiration (Fig. 3a). The forskolin-mediated increase in mitochondrial and uncoupled respiration were significantly higher in MMC-generated adipocytes compared to adipocytes differentiated with the cocktail alone (Fig. 3a iw v.s. iw MMC; Fig. 3b ib v.s. ib MMC). The greatest differences were observed between iw and ib MMC (Fig. 3c) which correlated to their relative levels of UCP1 expression.

### Micro-architectural effects of MMC mimics a native adipocyte ECM environment, which correlates with adipogenesis and *UCP1* expression through increased ECM engagement

**Figure 4:**
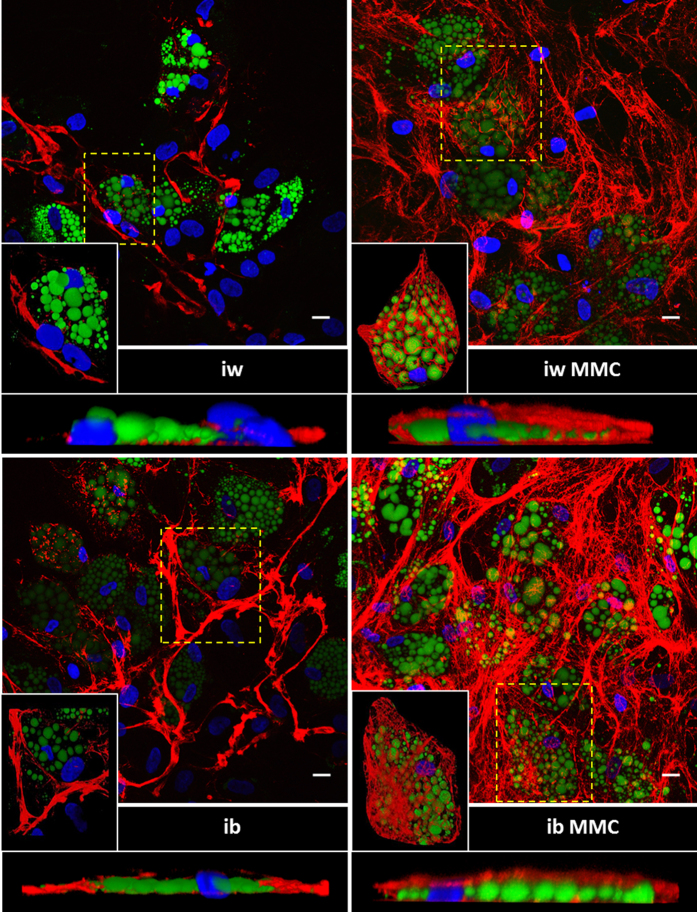
MMC enhances formation of a Col IV basement membrane architecture during adipogenic differentiation. Z-stack images were obtained for each condition through confocal microscopy. The Z-project images show nuclei (blue), Col IV (red), and lipid droplets (green). The inserts and cross-sections are the reconstructed 3D images of the selected cells boxed in yellow to show the pericellular distribution of Col IV. Scale bar: 20 μm.

**Figure 5:**
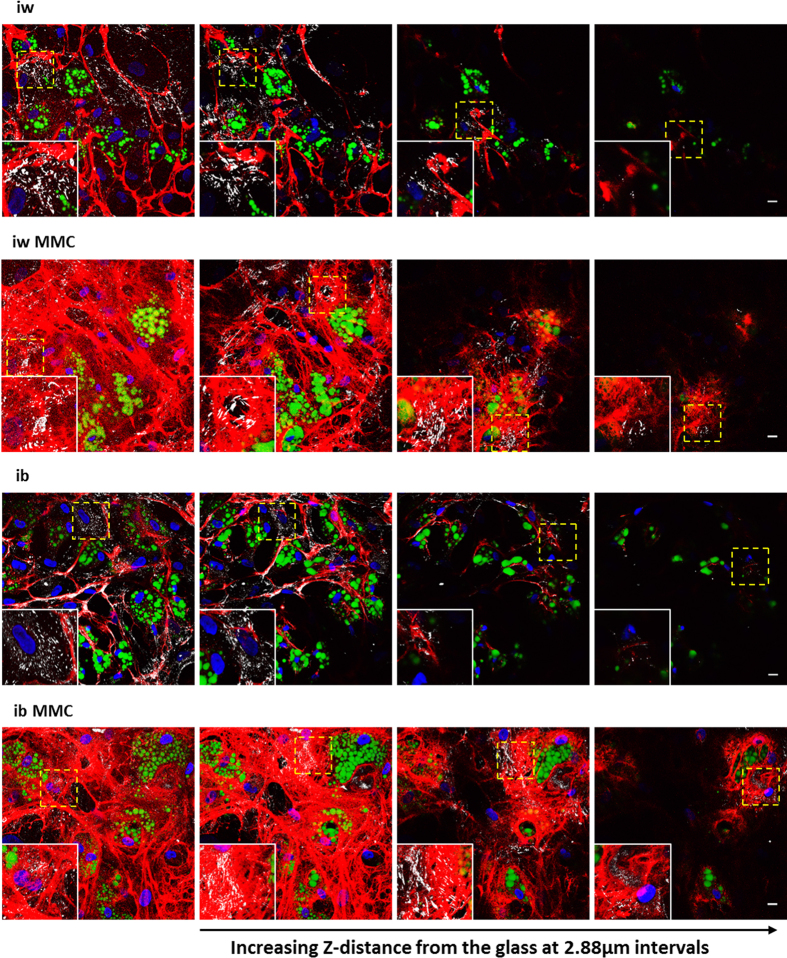
bmMSC-derived adipocytes under MMC show increased ECM engagement spatially. Z-stack images were obtained for each condition through confocal microscopy. Nuclei are depicted in blue, Col IV in red, lipid droplets in green and paxillin in white. Individual slices from the Z-stack are shown in increasing Z-distance from the glass coverslip per condition. Enlarged inserts are taken from the areas boxed in yellow to show clearly the paxillin staining. Scale bar: 20 μm.

**Figure 6:**
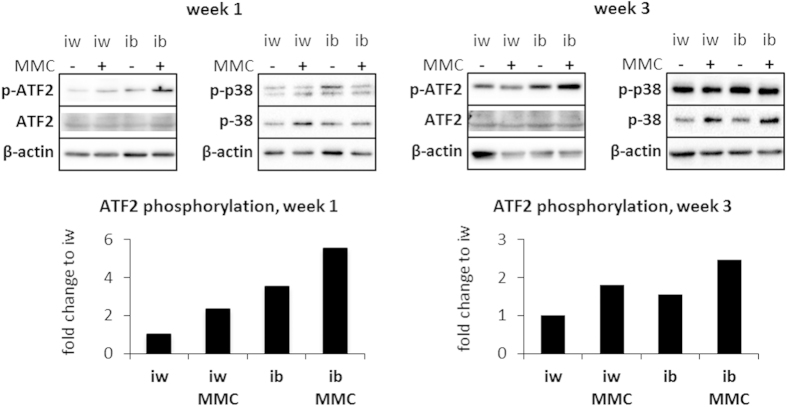
bmMSC-derived adipocytes under MMC show increased ATF2 activation during the 3-week time course of differentiation. Cell lysates were collected after 1 and 3 weeks of adipogenic differentiation. Western blotting for p-p38 MAPK, p38 MAPK and p-ATF2 were performed on the samples, with β-actin serving as the loading control. Quantification of the ATF2 phosphorylation was performed on these blots and are represented in the bar graphs. N = 1.

In corroboration of our earlier work^27^, quantitative analyses of whole bmMSC-derived adipocyte monolayers revealed a substantial increase of deposited collagen IV (Col IV) and heparan sulfate proteoglycans (HSPGs) under MMC (Fig. S5). A detailed confocal laser scanning analysis revealed that MMC did not increase the thickness, but the density of the Col IV deposited (Fig. S6) leading to fundamental architectural differences of Col IV distribution and microstructure under MMC. bmMSC-derived adipocytes differentiated in the absence of MMC generally demonstrated Col IV arranged in thick bundles running alongside adipocytes and undifferentiated bmMSCs alike (Fig. 4, left panel, insets). In contrast, MMC–treated cultures showed an abundant fine meshwork of Col IV fibres which enveloped the adipocytes, forming cocoon structures around individual cells, (Fig. 4, right panel) a feature observed *in vivo*^30^. MMC-treated cultures allowed for the formation of focal adhesions (visualized by paxillin) not only at the glass surface, but also around and on top of cells encased by Col IV. This suggested that the cells engaged the ECM from all spatial directions (Fig. 5, 2^nd^ and 4^th^ rows; Fig. S7). The spatial ECM engagement was lacking for cultures differentiated in the absence of MMC, as the formation of focal adhesions appeared restricted to contact sites with the glass surface (Fig. 5, 1^st^ and 3^rd^ rows). Western blotting of cell lysates showed increased paxillin content under MMC (Fig. S8), implying the formation of more focal adhesions compared to non-MMC conditions. To investigate whether the increased ECM engagement signaled UCP1 expression, the phosphorylation of p38 and its target ATF2 were assessed. While p-p38 was detected in weeks 1 and 3 of differentiation for all conditions, an increased ATF2 phosphorylation was observed in the MMC conditions in the 3^rd^ week of adipogenic differentiation (Figs 6 and S9). Moreover, after a 16 h forskolin stimulation, ATF2 phosphorylation was upregulated in the MMC conditions, i.e. iw MMC by 2.3-fold and ib MMC by 2.1-fold (Fig. S10). FGF21 mRNA, a downstream target of ATF2, was detected only in the MMC conditions (Table S1).

**Figure 7:**
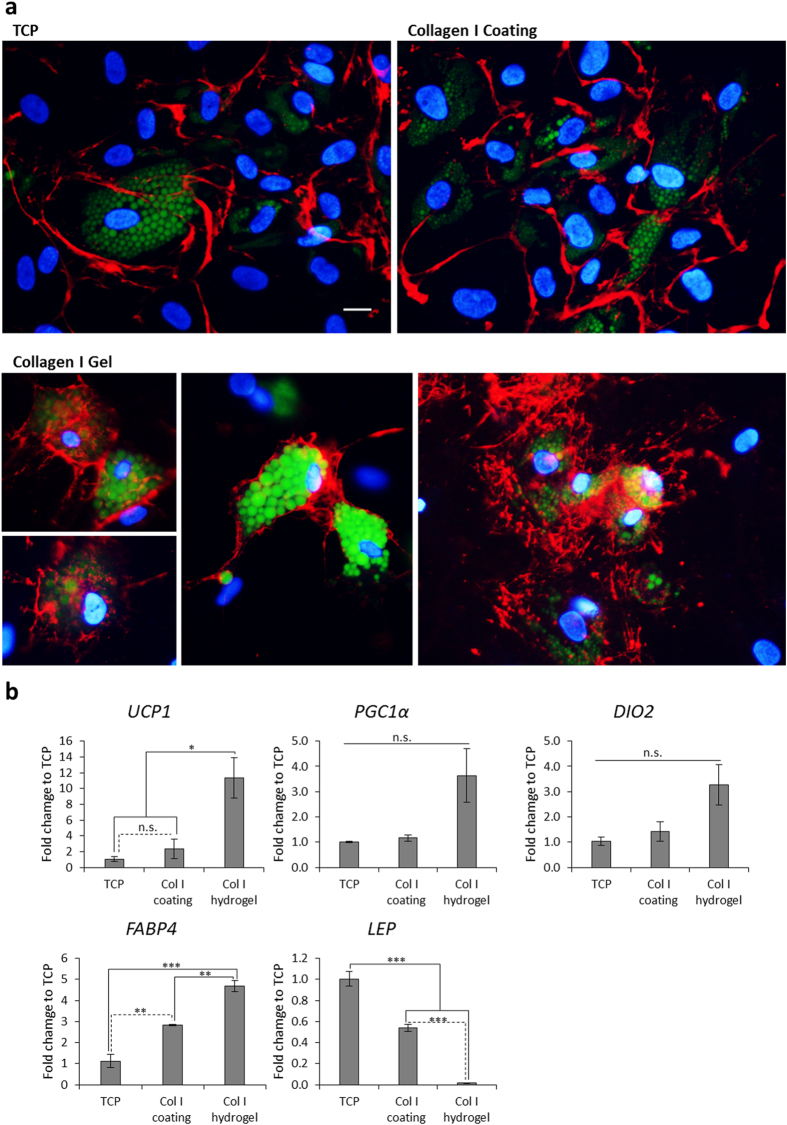
3D microenvironment promotes Col IV cocoon formation and brown adipogenesis in bmMSC-derived adipocytes. bmMSCs were differentiated for 2 weeks on TCP, bovine collagen I coating and hydrogel. (**a**) Fluorescence images of Col IV (red), lipid droplets (green) and DAPI-stained nuclei (blue) at 64× magnification. Scale bar: 20 μm. (**b**) qPCR analysis of: *UCP1* = uncoupling protein 1, *PGC1α* = PPARγ co-activator 1α, *DIO2* = deiodinase, iodothyronine, type II; *FABP4* = fatty acid binding protein 4, and *LEP* = leptin. Data is expressed as mean ± SEM. n.s. =not significant; *p < 0.05; **p < 0.01; ***p < 0.001. N = 3.

We then tested the concept of the effect of a microstructural arrangement as driving *UCP1* expression in the absence of MMC by differentiating bmMSCs with the iw induction protocol in a bovine collagen I hydrogel, thus providing a 3D microenvironment without MMC, and compared this with a collagen I coating (2D microenvironment) and tissue culture plastic (TCP). After 2 weeks, lipid-laden adipocytes were seen in all conditions. However, only the collagen I hydrogel conditions had distinct Col IV cocoon structures completely formed around individual adipocytes, whereas these structures were absent in the coating and TCP conditions (Figs 7a and S11). *UCP1* expression was significantly upregulated 5-fold in the hydrogel compared to the coating and 11-fold compared to TCP. The biological trend was similar for other brown adipocyte genes *PGC1α* and *DIO2* although there was no statistically significant difference between conditions. Pan-adipocyte marker *FABP4* was significantly upregulated the most in the hydrogel condition (5-fold) followed by the coating (3-fold), compared to TCP (Fig. 7b), while *LEP* was significantly down regulated the most in the hydrogel condition, showing an inverse correlation with the expression of brown adipocyte genes (Fig. 7b).

### MMC enhances browning of bmMSC-derived white adipocytes

**Figure 8:**
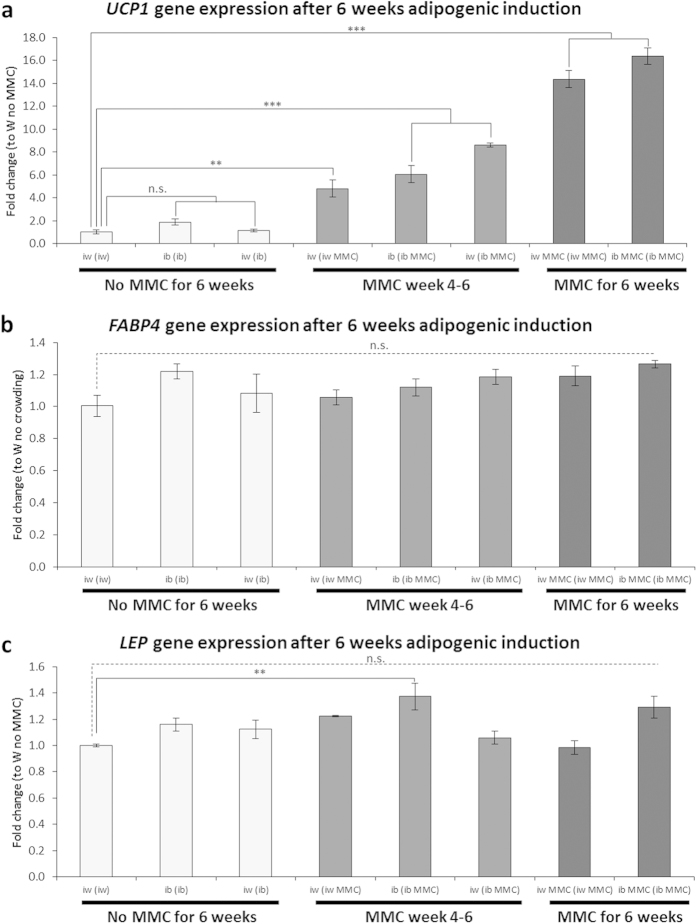
Browning of WAT-differentiated bmMSCs with MMC. bmMSCs were subjected for 6 weeks to either a white or brown protocol in the presence or absence of MMC. A third group of cultures was subject to a white or brown protocol for 3 weeks and then switched to MMC or a brown protocol with MMC. Conversion of white to brown was monitored by *UCP1* mRNA expression. (**a**) *UCP1*, (**b**) *FABP4* and (**c**) *LEP* expression were determined by qPCR. *UCP1* = uncoupling protein 1, *FABP4* = fatty acid binding protein 4, *LEP* = leptin. Data is expressed as mean ± SEM. n.s. =not significant; *p < 0.05; **p < 0.01; ***p < 0.001. N = 3.

To assess whether MMC could facilitate the ‘browning’ of bmMSC-derived white adipocytes, i.e. the emergence of brown adipocytes in a white adipocyte population, bmMSCs were first differentiated into white adipocytes for 3 weeks under the iw protocol and then switched to a ib cocktail ±MMC for another 3 weeks to assess the extent of browning (Fig. S12). At 3 weeks the adipocytes were deemed as mature as the levels of FABP4 did not change significantly between 3 and 6 weeks (Fig. S13a). bmMSC-derived adipocytes differentiated in either iw protocol and the ib MMC protocol for 6 weeks served as reference for baseline (1-fold) and maximal *UCP1* induction (16-fold), respectively (Fig. 8a). In the absence of MMC, neither the switch to the ib induction cocktail after 3 weeks nor a straight 6 weeks ib protocol induction led to a significant *UCP1* expression (1.2-fold, and 1.9-fold, respectively) (Fig. 8a, left column group). In contrast, the secondary addition of MMC in weeks 4–6 increased *UCP1* expression 4.8-fold in iw MMC, 6.1-fold (ib MMC) and 8.6-fold when the iw induction cocktail was switched to the ib induction cocktail (Fig. 8a, middle column group). 6 weeks of iw MMC induced cultures achieved 14-fold *UCP1*, closely approaching maximal *UCP1* induction values (16-fold) obtained for 6 weeks of ib MMC induction (Fig. 8a, right column group). Expression of pan-adipocyte genes *FABP4* and *LEP* was comparable to iw-generated adipocytes as a baseline (Fig. 8b,c), indicating that the upregulation of *UCP1* was not due to an overall enhanced adipogenesis (as in maturation) but reflected a browning effect of MMC. The 6 week experiment was re-performed using a different bmMSC lot and we found that the browning effect of MMC was preserved (e.g. a 5-fold increase when after 3 weeks, the induction cocktail was switched from iw to ib and MMC was added (iw (ib MMC))), albeit with more variation, probably due to the biological variance between the two bmMSC lots (Fig. S13b).

### Comparison of bmMSC with differentiation behavior of stromal vascular fraction (SVF) from abdominal subcutaneous (SC) fat reveals a mixed brown/brite phenotype

Abdominal SC adipose tissue has been used as a WAT control for human comparison studies of classical brown, beige and white fat (Jespersen *et al.*, 2013). However, SVF cells generated from this tissue readily responded to the ib cocktail in the absence of MMC (14-fold upregulation in *UCP1* compared to iw induction alone) (Fig. S14). MMC enhanced *UCP1* expression significantly by 33-fold in the ib MMC protocol (Fig. S14). Similar to bmMSCs, *PRDM16* expression did not change across conditions but *CIDEA* expression was significantly upregulated by 3-fold in the ib MMC condition. The brown marker *ZIC1* was well expressed stably across different induction conditions in bmMSCs, and not strongly in SVF (Fig. S15; Table S2). Complementarily, SVF strongly expressed brown marker *LHX8*, less so across all induction conditions, while bmMSCs showed negligible *LHX8* message. Brite markers *TMEM26* and *TBX1* showed mentionable expression only in bmMSCs, but in the case of *TMEM26* dropped under MMC. CD137 was lowly expressed in both bmMSC and SVF. The white marker *HOXC9* was well expressed in both cell sources and stably across all induction conditions with a larger abundance in SVF (Fig. S15; Table S2).

## Discussion

In the quest to establish a robust human cell model of brown and brite adipocyte physiology from an easily accessible and renewable cell source we have investigated the adipogenic potential of bone marrow derived mesenchymal stem cells. Here, we report that a functional brown/brite adipocyte potential can indeed be unlocked from this cell source. Using macromolecular crowding (MMC) as a biophysical inducer, we were able to differentiate – without the need of genetic manipulation – a functional BA phenotype from adult human bmMSCs, as characterized by the high expression of *UCP1*, increased mitochondrial membrane depolarization, oxygen consumption and uncoupled respiration upon thermogenic stimulation. The differentiation efficacy (defined as the level of *UCP1* expression benchmarked against a white differentiation protocol) in this cell model is comparable to other human cell models aiming at BA differentiation, including those based on induction of pluripotent cells^19^,^20^, human multipotent adipose-derived stem cells (hMADs) isolated from young donors^17^, and preadipocytes from the subclavicular region in human adults^15^.

Our differentiation system is robust; from four different lots of bmMSCs we have achieved predictable and stable phenotype conversion in at least 40 differentiation rounds and in the hands of different experimenters. Although widely accepted as indicative, the expression of UCP1 mRNA does not necessarily translate into thermogenic behavior^31^. To confirm this, we have demonstrated a marked increase in both oxygen consumption and uncoupled respiration in whole monolayer cultures of ib MMC-differentiated bmMSCs. Our findings highlight a surprising intrinsic potential of bone marrow derived mesenchymal cells for differentiation towards a brown fat phenotype. Interestingly, bone marrow fat has been referred to as yellow adipose tissue, and was traditionally seen as ‘space filler’ for areas no longer required for hematopoiesis. This simplistic view has been evolving with the suggestion that marrow fat might play potential roles in regulating haematopoiesis^32^, possibly osteogenesis, and most notably, systemic energy metabolism^33^. Moreover, it has recently been speculated that bone marrow may harbor a mixed population of white and brown fat phenotypes^20^,^34^,^35^,^36^. Under a white induction protocol, isolates of fetal bmMSCs have been observed to occasionally differentiate *in vitro* into BA^37^, while other work suggests that human adult bmMSCs can differentiate only into white adipocytes under these conditions^21^. However, adenovirus-mediated forced overexpression of PGC1α in adult bmMSCs can elicit a moderate increase in *UCP1* expression with a 2-fold increase in mitochondrial mass and oxygen consumption^38^ suggesting that a latent brown potential persists in bmMSCs. We believe that this somewhat confused literature on brown progenitors in bone marrow can be reconciled by our data, which suggest that adult bmMSCs do indeed represent a mixture of both white and brown progenitors. The brown potential of these progenitors, however, appears to be suppressed under routine monolayer and aqueous (non-crowded) standard culture conditions.

Similarly, with MMC we could differentiate progenitors from the SVF fraction of SC abdominal fat into BA. The SC fat depot, has been considered “white”, but has also been shown to harbor inducible brown/brite (brown in white) adipocytes recognizable under certain induction conditions^39^,^40^,^41^. While both bmMSCs and the SC abdominal WAT SVF represent heterogeneous cell populations (in both cell cultures a certain percentage of cells did not differentiate to adipocytes) the SVF cultures appeared more responsive to the biochemical brown induction (ib) protocol, even in the absence of MMC. This would seem to be in agreement with very recent beige differentiation work using either prolonged BMP7 exposure^42^ in combination with a brown differentiation cocktail, or a 32-day protocol of continued induction with a brown differentiation cocktail alone^43^. In both cases the drug rosiglitazone was used, and UCP1 mRNA induction levels of 15-fold after a total of 10 days and up to 280-fold after 32 days were reported, respectively. We could confirm the thermogenic potential of SVF under ib conditions in a 24-day protocol including BMP7 pretreatment^42^,^43^. However, brown induction in SVF was greatly augmented in the presence of MMC. In essence, both SVF and bmMSCs represent perivascular stromal cells of mesenchymal origin. PRDM16 and CIDEA have been classified as brown-selective markers as they are highly expressed in mature BAT compared to WAT in mice. PRDM16 was described first in mice as a transcription factor deciding between myocyte and “classical” brown adipocyte fate in myf5+ embryonic mesenchymal progenitor cells^44^. It may not be crucial in brite adipocytes to confer the ability to express UCP1^2^. In our hands, human bmMSCs- and SVF- derived BA (ib MMC) expressed PRDM16 appreciably across all conditions; however, also non-induced bmMSCs expressed PRDM16 (not shown). Therefore, PRDM16 might indicate a brown potential in cells not expressing high abundance copies of UCP1 at a given time point.

We applied a current gene marker panel for distinguishing white, brite and classical BA that has been recently validated in mouse and human tissues^3^,^7^,^10^. While there was a clear indication of brown markers *ZIC1* for bmMSCs, and *LHX8* for SVF, respectively, we also noted the moderate expression of brite markers in bmMSCs. In addition, the white marker *HOXC9* was robustly expressed, but remained unchanged across all induction conditions and with both cell sources. Since both cell types do not come from pure bona fide BAT deposits, the UCP1 expressing cells we generate would, by definition, be classified as brown-in-white (brite). As noted, brite markers were expressed in bmMSCs, but not in the SVF populations where we might have expected them^42^,^43^. In addition, each cell type expressed a different classical brown marker. We conclude therefore that both cell sources contain a mixture of white adipocytes, of which a sub-population is ‘brownable’, and a genuine brown adipocyte progenitor population. A potential caveat to this argument is the functional ‘provenance’ of these markers^45^. With the exception of UCP1, it is possible that the current panel of brown/brite markers for human progenitor cells may not be truly indicative of thermogenic potential, but rather reflective of the anatomical tissue location from which they were isolated.

In addition to revealing the brown potential of human mesenchymal progenitors, we demonstrate how this potential can be effectively realized *in vitro*. Tuning the microenvironment of these cells in monolayer cultures can greatly assist in pushing them across the differentiation threshold. The mechanism of this process can be potentially explained at several levels of cell-matrix interaction. Firstly, the enhanced matrix produced by MMC-treated differentiating adipocytes may sequester more growth factors due to the increased amount of HSPG^46^, which in turn would further the differentiation process. Secondly, the niche-specific composition and structure of the ECM influences cell behavior, growth, fate and migration through orchestrated interactions with various cell surface receptors. *In vivo*, adipocytes are enveloped by a pericellular basement membrane composed largely of Col IV which surrounds the adipocyte^30^. This pericellular structure is important to the adipocyte phenotype, as inhibition of its formation impairs adipogenic differentiation^30^. We have recently shown that macromolecular crowding drives and augments extracellular matrix deposition^26^, and influences collagen hydrogel architecture leading to the formation of more, but finer individual collagen fibers^47^. In addition, recent in silico work has explained the potentiating effects of mixed MMC^48^. We therefore predicted comparable micro-architectural changes in cell culture brought about by mixed MMC. Indeed, high resolution imaging confirmed that adipogenic differentiation of bmMSCs under MMC yields a distinct ECM architecture whereby individual adipocytes are encased in a basket of Col IV fibres, similar to what has been observed *in vivo*^49^. In comparison, adipocytes induced without MMC (standard culture) not only deposit less Col IV but also assemble it into thick coarse fibres running along the basolateral periphery of the cells but not encasing them. The pericellular cocoon of Col IV formed under MMC provides a 3D environment for increased cell-matrix interactions. This was evidenced by the increased amount of focal adhesions that formed all over the cell surface including the apical side of the cells. Focal adhesions have been well described in monolayer “2D” environments, exclusively on the underside of cells at sites of contact with the support structure. The existence of focal adhesions in 3D environments has been debated^50^, but later was demonstrated in hydrogel systems^51^. Our data strongly suggest that focal adhesions develop in a 3D ECM microenvironment generated under MMC in monolayer culture. This is remarkable, as it was originally reported that only stiff substrates promote focal adhesion growth and elongation^52^. This feature was seen as guiding cells into the osteogenic lineage. Here, we show that a total ECM encasing of bmMSCs in a monolayer increases the 3D distribution of focal adhesions, and that this constellation correlates with increased adipogenesis. The spatial distribution of focal adhesions signifies also a multidirectional engagement of ECM; here, cells can engage the Col IV scaffold directly or indirectly via Col IV ligands such as laminin^53^,^54^ through interaction with integrin α6, thus enhancing adipogenic differentiation^53^,^55^. To further illustrate the importance of the 3D Col IV encapsulation in promoting UCP1 expression, we compared the differentiation of bmMSCs on a 2D coating and a 3D hydrogel of collagen I respectively and found that only the 3D hydrogel had the individual adipocytes wrapping themselves in a Col IV cocoon while significantly expressing UCP1. The striking similarities between the Col IV cocoon formation in monolayer under MMC and in the collagen I hydrogel (without MMC), and the concomitant increase in UCP1 expression in both cases strongly suggest that it is the micro-architectural effects of MMC that drives UCP1 expression.

**Figure 9:**
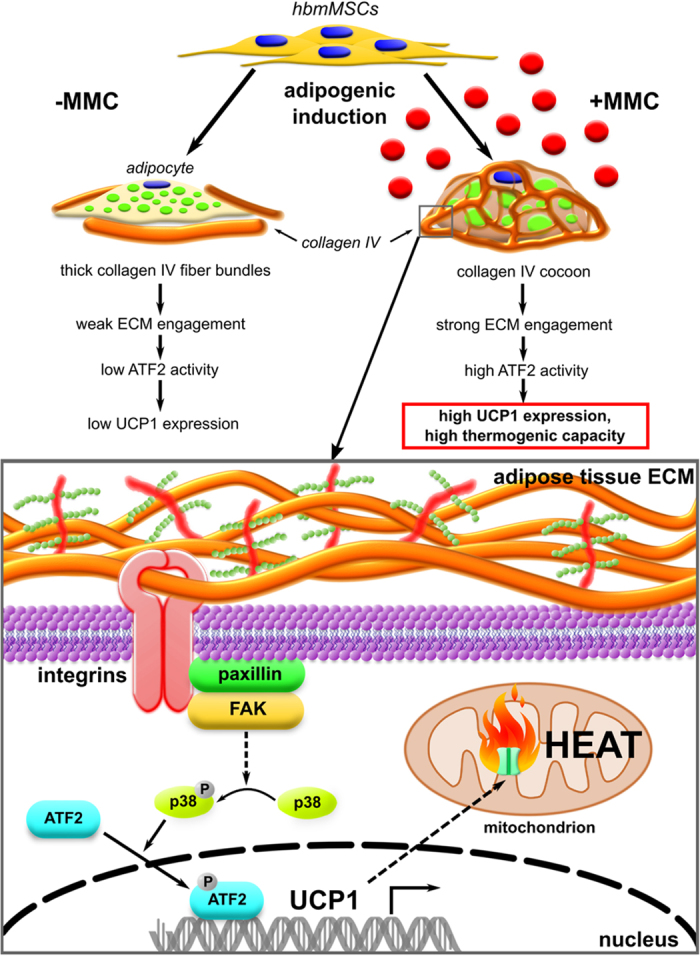
Representative diagram of MMC-enhanced Col IV formation driving brown adipogenesis in bmMSC-derived adipocytes.

Thirdly, we and others have shown that adipogenesis is accompanied not only by synthesis, but also by proteolytic remodelling of Col IV secreted during the course of differentiation in cell culture by matrix metalloproteinases^27^,^56^. The resulting, partially denatured Col IV, can promote differentiation of bmMSCs into fat cells^57^. It was speculated that the exposure of cryptic integrin α_V_β_3_ binding sites upon Col IV denaturation activates p38 MAPK signaling^58^ which in turn promotes adipogenesis^59^, and stimulates UCP1 transcription through the activation of transcription factor ATF2^60^. In our system, p38 MAPK was active throughout the course of adipogenic differentiation but phosphorylated p38 MAPK levels were not increased under MMC. This could be due to the timing of our observation that only began after one week and thus transient p38 MAPK activation peaks might have been missed. In addition, p38 MAPK activity is tightly regulated in cells, as overstimulation would potentially lead to cell death^59^. However, significant levels of p-ATF2 were observed in bmMSC-derived adipocytes induced under MMC. This finding delineates the possible mechanistic loop between MMC-modulated microenvironment formation, matrix-cell signaling and UCP1 induction (Fig. 9), the details of which will be subject to future work.

Lastly, and perhaps most significantly, MMC using the sucrose co-polymer Ficoll also encourages the conversion of adult human bmMSC-derived white adipocytes into a brown/brite phenotype with a 16-fold increase in *UCP1* expression, again without genetic manipulation, an efficiency comparable to the only other white-to-brown conversion model in human progenitor cells from prepubic fat of infants (hMADs) and a PPARγ agonist^17^. The evolution of the white differentiation state between weeks 3 and 6 in the iw condition (assessed by FABP4 expression) did not change, which suggests that the preadipocytes have been recruited to form white adipocytes at 3 weeks and these white adipocytes convert to brown/brite adipocytes under MMC. This potential is consistent with the suggestion that marrow fat retains the plasticity to transdifferentiate from white adipocytes to a population of UCP1-expressing adipocytes.

The current study has revealed the intrinsic potential of the bone marrow to harbor a novel population of brown adipocyte progenitors not previously described. The capacity to generate BA from bmMSCs was found to be dependent on the microenvironment which was dimensionally shaped by MMC. We believe that our data support new opportunities for isolating and manipulating adult human mesenchymal progenitor cells, as a robust, autologous and renewable source of BA. Such cells will make technology platforms possible for nutraceutical and pharmacological screening, and shall open avenues for personalized medicine in a bid to address clinical issues of metabolic syndrome and related diseases.

## Methods

### Bone marrow mesenchymal stem cell culture

Adult human bone marrow-derived mesenchymal stem cells (bmMSCs) were obtained commercially from Lonza (PT-2501), and expanded in DMEM supplemented with GlutaMAX^TM^, 10% FBS and penicillin (100 units/ml)/streptomycin (100 μg/ml) (P/S) (10567, 10270 and 15140, Life Technologies, Carlsbad, CA, USA). Four different lots were used in this paper.

### Adipose tissue biopsies

Subjects recruited were candidates who opted for bariatric surgery and were ≥21 years old, obese with body mass index (BMI) ≥35 kg/m^2^. We excluded those with diabetes mellitus (defined as HbA1c > 6% or fasting glucose >6.0 mmol/l), a history of malabsorption syndrome, Crohn’s disease, ulcerative colitis, pancreatitis, and a history of ingesting drugs known to alter insulin sensitivity (e.g., corticosteroids). All subjects had provided informed consent and were patients from the Centre for obesity and metabolic surgery, National University Health System (NUHS, Singapore). A detailed medical history was collected from each subject and this included the baseline anthropometric measurements, complete medical history, physical examination, blood pressure measurement, presence of other cardiovascular disease risk factors (e.g., hypertension, dyslipidemia, smoking, and alcohol intake), presence of albuminuria, and all existing medications prescribed. Adipose tissue specimen was obtained from the subcutaneous abdominal area towards the end of the bariatric surgery, without any additional surgical procedure. Ethics approval was obtained from the National Healthcare Group Domain Specific Review Board (Singapore) and the procedures were carried out in accordance with the approved guidelines.

### Isolation of progenitor cells from the adipose stromal vascular fraction (SVF)

Freshly isolated adipose tissue was processed as described previously^61^ with some modifications. Briefly, adipose tissue was minced and digested with 1 mg/ml collagenase (C5138, Sigma-Aldrich, St. Louis, MO, USA) and 2% bovine serum albumin (BSA) (A9418, Sigma-Aldrich) for 1 h in a 37 ^o^C incubator shaker. Digested tissue was filtered through 100 μm cell strainer and centrifuged at 400 g for 5 min. Stromal vascular pellet was re-suspended in 10 ml Hank’s balanced salt solution (HBSS) (14175, Life Technologies) and subjected to centrifugation and washed for three times. The remaining pellet was subsequently re-suspended in 10 ml DMEM (11995, Life Technologies) containing 15% FBS (SV30160.03, Thermo Fisher Scientific), 1% non-essential amino acids (11140, Life Technologies), 1 μg/ml human recombinant basic fibroblast growth factor (PHG0021L, Life Technologies) and P/S (100 units/ml)/(100 μg/ml) and incubated in a 10 cm petri dish at 37 ^o^C for 1 h. Finally, the non-adherent cells, containing adipose-derived stromal cell population, were transferred to a T75 culture flask. The progenitor cells were deemed ready for differentiation experiments when they displayed a fibroblastic morphology.

### Adipogenic induction of human mesenchymal adipogenic progenitor cells

bmMSCs or the progenitor cells from the adipose SVF were seeded at an initial density of 10.5 k cells/cm^2^. For oxygen consumption measurements, bmMSCs were seeded at 10 k/well in the XF24 cell culture plates (100850-001, Seahorse Bioscience, Chicopee, MA, USA). Adipogenic differentiation was stimulated when the cells reached confluence as described^22^,^62^ (with modifications) via three cycles of 4 days of induction, followed by 3 days of maintenance. Non-induced controls were maintained with basal media alone on the same schedule. The basal media used in the differentiation process composed of high glucose DMEM supplemented with GlutaMAX^TM^, 10% FBS (10569, 10270, Life Technologies) and P/S (100 units/ml)/(100 μg/ml). The white induction (iw) cocktail consisted of 0.5 mM 3-isobutyl-1methylxanthine (IBMX), 0.2 mM indomethacin, 1 μM dexamethasone and 10 μg/ml insulin (I5879, I7378, D4902, 91077C, Sigma-Aldrich). Basal media alone was used during the maintenance phase. In the ib induction protocol, cells were pre-treated with 125 ng/ml bone morphogenetic protein 7 (BMP7) (354-BP, R&D Systems, Minneapolis, MN, USA) 3 days prior to differentiation. The brown induction (ib) cocktail consisted of the iw cocktail with the addition of 1 nM triiodothyronine (T3) (T5516, Sigma-Aldrich) and 1 μM rosiglitazone (ALX-350-125, Enzo Life Sciences Inc., Farmingdale, NY, USA). Basal media alone was used during the maintenance phase. For conditions treated with macromolecular crowding (+MMC), the media was supplemented with a mixture of Ficoll^TM^70 at 37.5 mg/ml and Ficoll^TM^400 at 25 mg/ml (17-0310, 17-0300, GE Healthcare, Bio-sciences AB, Uppsala, Sweden), throughout the differentiation process. Media were sterile filtered prior to use. For thermogenic stimulation, 10 μM forskolin (F6886, Sigma-Aldrich) was used. Unstimulated cultures were incubated for the indicated period with either only basal media or with the addition of an equal amount of DMSO as a vehicle.

### Browning of bmMSC-derived white adipocytes with MMC

To assess whether mature bmMSC-derived white adipocytes could be ‘browned’ in culture, bmMSCs were first subjected to a standard white (iw) protocol for 3 weeks. For the next 3 weeks, these cultures were either maintained in iw conditions or switched to ib cocktail in the absence or presence of MMC. Various combinations of iw and ib cocktails in the absence or presence of MMC served as controls to assess the browning effect of the cocktail or MMC alone, generating a total of 8 conditions (Fig. S12). bmMSCs differentiated for 6 weeks straight in iw and ib MMC served to define the bandwidth between baseline and maximal UCP1 induction respectively.

### Visualization and quantitation of lipid droplet content

Cell cultures were rinsed with PBS, fixed in 4% formaldehyde for 30 min at RT, then co-stained for 30 min with 5 μg/ml Nile Red (N3013, Sigma-Aldrich) for lipid droplets and 0.5 μg/ml of 4′,6-diamidino-2-phenylindole (DAPI) (D3571, Life Technologies) for nuclear DNA. Adherent cytometry was performed according to a previously described protocol^27^. Extent of adipogenic differentiation was quantified by area of Nile Red fluorescence normalized to nuclei count (μm^2^/nuclei).

### Quantitative PCR

Total RNA extracted from monolayers was reverse-transcribed and real time quantitative PCR using Maxima™ SYBR Green/ROX qPCR Master Mix (K0222, Thermo Fisher Scientific) was performed for 35 cycles as previously described^27^. Relative gene expression levels were determined using the ΔΔ-Ct method. Results were normalized to either TATA-box binding protein (TBP) or the geometric mean of human ribosomal phosphoprotein P0 (RPLP0) and TBP. Primer sequences are given in Table S3.

### Quantification of mitochondrial mass

After 3 weeks of adipogenic differentiation, cells were subjected to 16 h forskolin stimulus or DMSO as vehicle control. Cells were then stained live with 200 nM Mitotracker Green (M7514, Life Techonologies) and quantified by adherent cytometry and as previously described^27^. Mitochondrial mass was quantified by area of fluorescence normalized to nuclei count (μm^2^/nuclei), where the nuclei were counterstained with DAPI after the mitotracker Green quantification on fixed cells.

### Western blotting

Sample protein extracts were obtained from cell monolayers and were separated using SDS-PAGE under reducing conditions and then transferred onto a nitrocellulose membrane (Bio-Rad, Hercules, CA, USA). For immune-detection, primary antibodies used were: anti-mouse UCP1 (1:1000, mab6158, R&D Systems, Minneapolis, MN, USA), anti-mouse paxillin (1:200, 3127, Abcam, Cambridge, UK), anti-rabbit p38 (1:1000), anti-rabbit p-p38 (1:1000), anti-rabbit p-ATF2 (1:1000) (9212, 9211, 9225, Cell Signalling, Danvers, MA, USA), anti-mouse ATF2 (1:1000, WH0001386M2, Sigma-Aldrich) and anti-mouse β-actin (1:1000, A2228, Sigma-Aldrich) as a loading control. Bound primary antibody was detected with HRP goat-anti mouse or HRP goat-anti rabbit antibodies (P0447, P0448, Dako, Glostrup, Denmark). Chemiluminescence was captured with a ChemiDoc MP Imaging System (Bio-Rad). Quantification of the ATF2 phosphorylation was performed on the blots by normalizing the intensity of p-ATF2 and ATF2 to its corresponding β-actin and then dividing the normalized value of p-ATF2 by ATF2. The values are represented in bar graphs as a fold change to the iw condition. Intensity measurements were obtained using ImageJ software (NIH).

### Determination of membrane potential by JC-1

Cells were washed with PBS and incubated with 5 μM JC-1 (T3168, Life Technologies) for 15 min at 37 ^o^C. Cells were then washed with PBS and stimulated with 10 μM forskolin or DMSO for 4 h before images were captured using the Olympus FluoView™ FV1000 confocal microscope (Olympus, Center Valley, PA, USA). Images were analysed using ImageJ software.

### Measuring mitochondrial oxygen consumption

Cells were incubated in 500 μl of XF assay medium (Seahorse Bioscience) for ~30 min at 37 °C in a warming chamber at atmospheric conditions before loading into the Seahorse XF24e analyser to measure the oxygen consumption rate (OCR). 10 μM Forskolin (or DMSO as vehicle control) was added to stimulate the cells for 100 min. Oligomycin (2 μM), FCCP (0.6 μM), and rotenone (2 μM) plus antimycin A (2 μM) from the XF Cell Mito Stress Test Kit (Seahorse Bioscience) were then added sequentially to dissect the different components contributing to total oxygen consumption. The calculations for mitochondrial respiration = OCR just before the addition of oligomycin; uncoupled respiration = the minimum OCR after the addition of oligomycin before FCCP; The minimum OCR after the addition of rotenone and antimycin A were subtracted from these values to account for non-mitochondrial respiration.

### Immunocytochemistry and quantification of extracellular matrix (ECM) deposition

Monolayers were fixed with 4% formaldehyde for 10 min at RT, then blocked with 3% BSA in PBS for 1 h. Immunofluorescence was carried out using the following primary antibodies: rabbit anti-human Col IV (1:500), mouse anti-human heparan sulfate proteoglycan 2 [A76] (HSPG) (1:200), mouse anti-human paxillin (1:200) (6586, 26265, 3127, Abcam). Primary antibodies were incubated overnight at 4 °C. Secondary antibodies used were AlexaFluor® 488 chicken anti-rabbit, 594 goat anti-rabbit, 594 goat anti-mouse and 647 goat anti-mouse (A21441, A11072, A11020, A21235, Life Technologies) at 1:400 dilution, incubation of 1 h at RT. Cell nuclei were counterstained with 0.5 μg/ml DAPI and cytoplasmic lipids with 0.1 μg/ml BODIPY 493/503 (D3922, Life Technologies). Images were either captured with an IX71 inverted fluorescence microscope (Olympus) or with a confocal laser scanning microscope (LSM510, Zeiss, Germany). Deposition of Col IV and HSPG were quantified by adherent cytometry and as previously described^27^. Extent of ECM deposition was quantified by area of fluorescence normalized to nuclei count (μm^2^/nuclei).

### Confocal laser scanning microscopy

Image acquisition was performed with a confocal laser scanning microscope (LSM510, Zeiss, Germany) with an EC Plan-Neofluar 40×/1.30 Oil objective. The pinhole was set at 74 μm. The emission filters used were BP 420–480 for the blue channel, BP 505–550 for the green channel, BP 575–615 IR for the red channel and LP 650 for the Far-red channel. The beam splitters used were HFT 405/488/561 (MBS), NFT 565 (DBS1), NFT 490 (DBS2), for the green and blue channels. For the red channel, the beam splitters used were HFT 405/488/561 (MBS), NFT 565 (DBS1) and Plate (DBS3). For the Far-red channel, the beam splitters used were HFT 405/488/633 (MBS), NFT 635 Vis (DBS1) and Plate (DBS3). The Lasers used were Diode 405 nm (blue) at 55.0%, Argon/2 (green) at 5.0%, DPSS 561 nm (red) at 1.1% power, and HeNe 633 nm (Far-red) at 30.0%. The acquisition of successive images in the Z (vertical) axis was performed by using the Z-stack application in the ZEN software (2009) with the interval between 2 planes set at 0.48 μm. The 3D rendering was performed by first converting the lsm images from the Z-stacks into TIFF images using the ZEN software. The pictures were then imported in the ImageJ software (File ->Import ->Image sequence) and treated by the 3D module (Plugins ->3D ->3D viewer).

### Quantitation of Col IV from the Z-stack images

Images of the Col IV staining were processed using ImageJ software to remove background noise and calculate the average pixel intensity per confocal plane, which was then plotted against the Z-distance (μm) for each image stack. Details on the calculations for the thickness and density of the Col IV matrices are given in the supplementary information.

### Collagen hydrogel studies

To prepare the hydrogels, soluble collagen I from bovine dermis at 3 mg/ml (IAC-30, KOKEN Cosmo Bio Co. Ltd., Japan) was diluted in acetic acid (0.05 M) and neutralized using NaOH (0.15 M) in PBS on ice to a final concentration of 0.8 mg/ml as previously described^48^. The hydrogel solution was then pipetted into well plates at 133 μl per 48-well and 350 μl per 24-well and the plates incubated at 37 ^o^C for polymerization. To prepare the coatings, the soluble collagen I stock solution (3 mg/ml) was added directly to the well plates at 50 μl per 48-well and 100 μl per 24-well and left to air-dry at RT for 3 h then 37 ^o^C for 45 min. Excess solution was removed and wells were rinsed with PBS, resulting in a thin coating of polymerized collagen. Cells were then seeded on TCP, coating and hydrogel at 18 k/cm^2^. Differentiation using the iw induction protocol was started the next day. Cultures were harvested after 2 weeks of differentiation for imaging and qPCR.

### Statistical analyses

Student’s t-test was used for comparison between two groups. For multiple pairwise comparisons, one-way ANOVA statistical analysis was performed followed by Tukey-Kramer Multiple Comparisons Test using Graphpad InStat software (GraphPad Software, Inc., CA, USA).

## Additional Information

**How to cite this article**: Lee, M. H. *et al.* ECM microenvironment unlocks brown adipogenic potential of adult human bone marrow-derived MSCs. *Sci. Rep.* **6**, 21173; doi: 10.1038/srep21173 (2016).

## Supplementary Material

Supplementary Information

Supplementary video 1

Supplementary video 2
