# Supplementary material for: ECM microenvironment unlocks brown adipogenic potential of adult human bone marrow-derived MSCs
Source: Sci Rep. 2016 Feb 17;6:21173. doi: 10.1038/srep21173 (PMC4756694; doi:10.1038/srep21173)
Supplement: Supplementary Information [file srep21173-s1.pdf]

SUPPLEMENTARY INFORMATION

ECM microenvironment unlocks brown adipogenic potential of adult human bone marrow-derived MSCs

Michelle H. Lee[1,2,3,+], Anna G. Goralczyk[1,3,+], Rókus Kriszt[1,2,3], Xiu Min Ang[1,2,3], Cedric Badowski[4], Ying Li[5], Scott A. Summers[6], Sue-Anne Toh[7,8], M. Shabeer Yassin[8], Asim Shabbir[9], Allan Sheppard[10], and Michael Raghunath[1,3,11,*]

[1]Department of Biomedical Engineering, National University of Singapore, 117575, Singapore
[2]NUS Graduate School for Integrative Sciences and Engineering (NGS), National University of Singapore, 117456, Singapore
[3]NUS Tissue Engineering Program, Life Science Institute, National University of Singapore, 117510, Singapore
[4]Institute of Medical Biology, A*STAR, 138648, Singapore
[5]Program in Cardiovascular and Metabolic Diseases, Duke-NUS Medical Graduate School, 169857, Singapore
[6]Translational Metabolic Health Laboratory, Baker IDI Heart and Diabetes Institute, Melbourne VIC 3004, Australia
[7]Department of Medicine, National University Health System, 119228, Singapore
[8]Department of Medicine, Yong Loo Lin School of Medicine, National University of Singapore, 117599, Singapore
[9]Department of Surgery, National University Hospital, 119074, Singapore
[10]Liggins Institute, University of Auckland, Auckland 1142 New Zealand
[11]Department of Biochemistry, Yong Loo Ling School of Medicine, National University of Singapore, 117599, Singapore

+MHL and AGG share first authorship
*Correspondence: bierm@nus.edu.sg

Supplementary Data

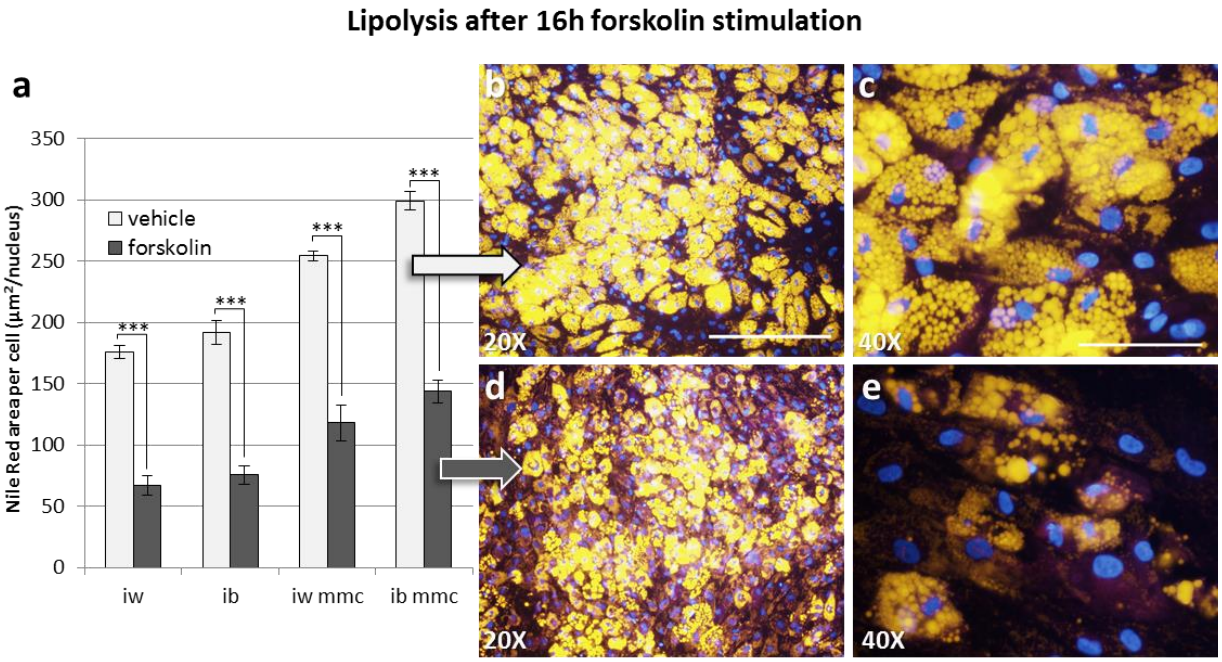

Figure S1. bmMSC-derived adipocytes undergo lipolysis after a forskolin stimulus. Adipocytes that were differentiated from bmMSCs were subjected to a forskolin (10µM) stimulus, and lipolysis was assessed by quantifying the loss of lipid stores. (a) Extent of lipolysis after a 16h forskolin treatment as captured by adherent cytometry quantifying Nile Red-positive area normalized to nuclei count. (b and c) vehicle treatment only, (d and e) 16h forskolin. c = non-induced control; iw = white cocktail; ib = brown cocktail. Scale bar for 20X: 200µm. Scale bar for 40X: 100µm.

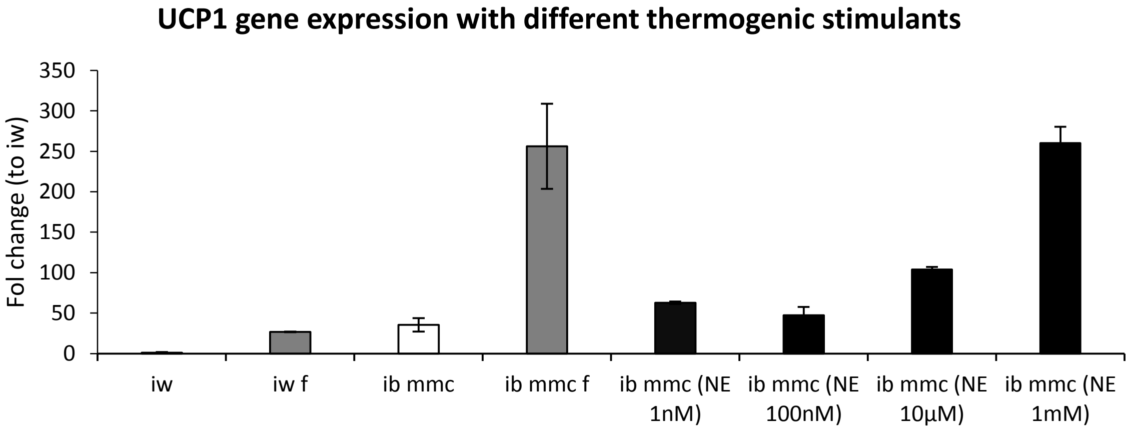

Figure S2. bmMSC-derived adipocytes (ib mmc) respond to norepinephrine (NE) by upregulating *UCP1* expression. Adipocytes that were differentiated from bmMSCs were subjected to a forskolin (10µM) or NE stimulus for 4h, and UCP1 gene expression was determined by qPCR. Duplicates were used in this experiment.

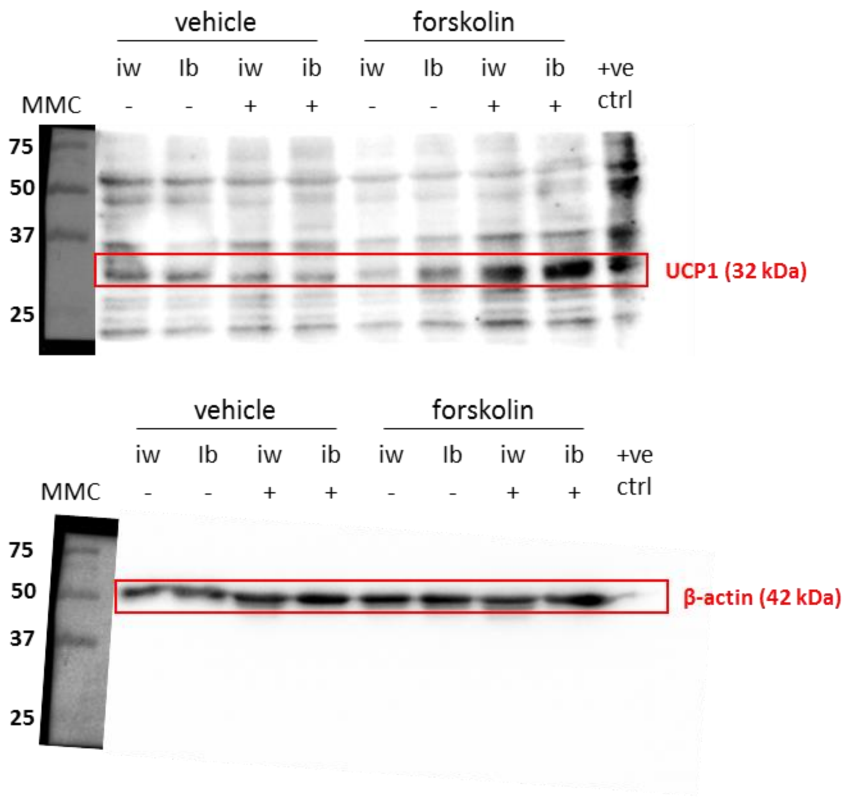

Figure S3. Uncropped UCP1 western blot of figure 2b. Differentiated murine brown pre-adipocyte cell line WT-1 was used as a positive control.

a

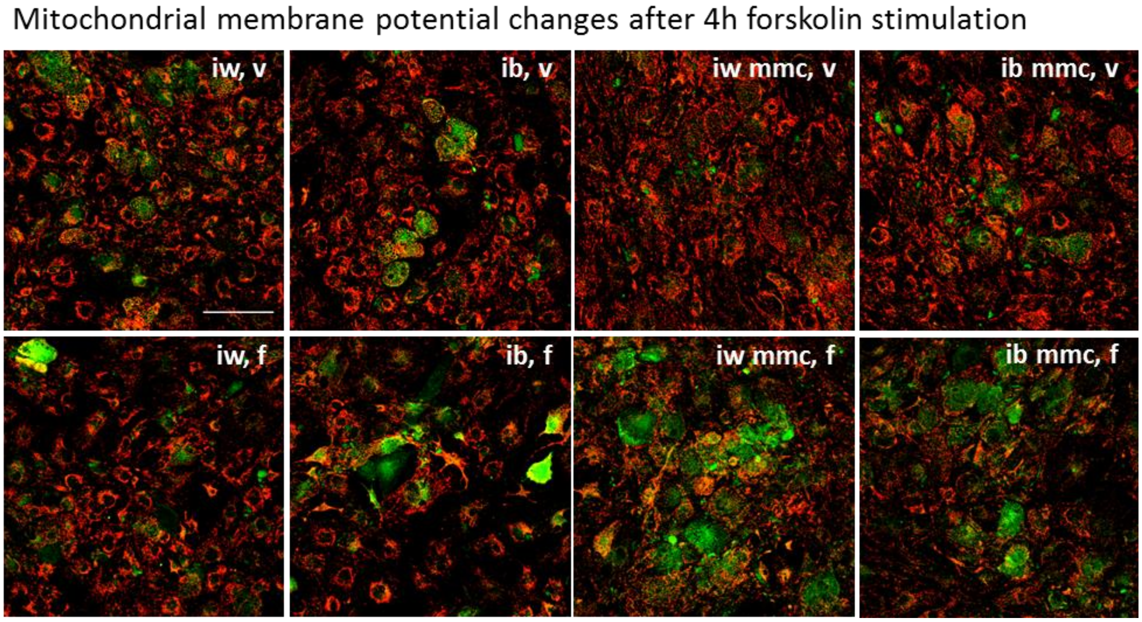

b

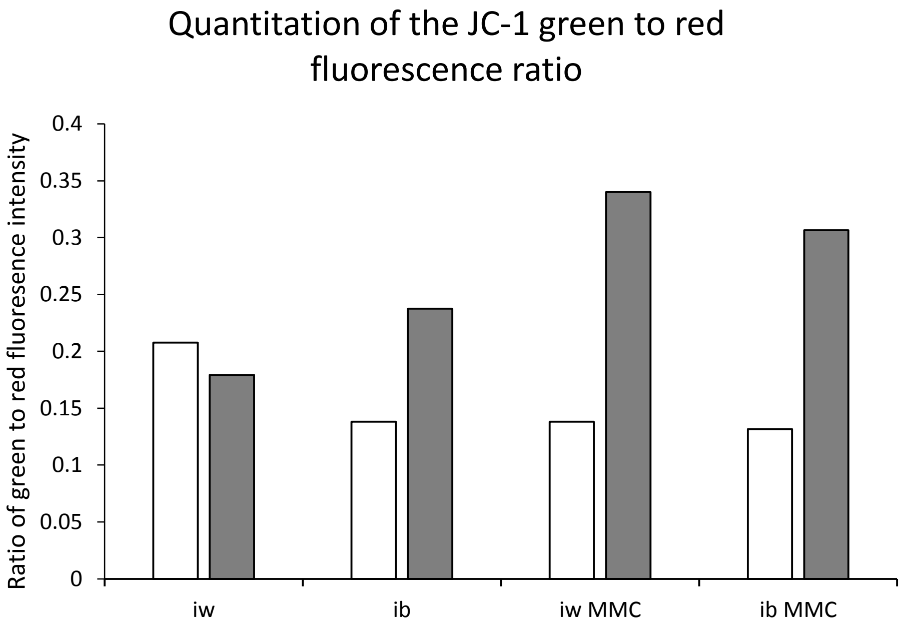

Figure S4. Mitochondrial membrane depolarisation in bmMSC-derived adipocytes after 4h forskolin stimulation. bmMSCs of a different lot from figure 2 were differentiated into adipocytes ±MMC and subjected to forskolin (10μM) for 4h. Mitochondrial membrane depolarization, an indication of uncoupled respiration, was assessed using JC-1. (a) Confocal images of vehicle-treated (top panel) and forskolin treated (bottom panel) adipocytes. (b) Quantification of the ratio of green to red fluorescence intensity of the images in (a), depicting the extent of depolarization. Scalebar: 200μm.

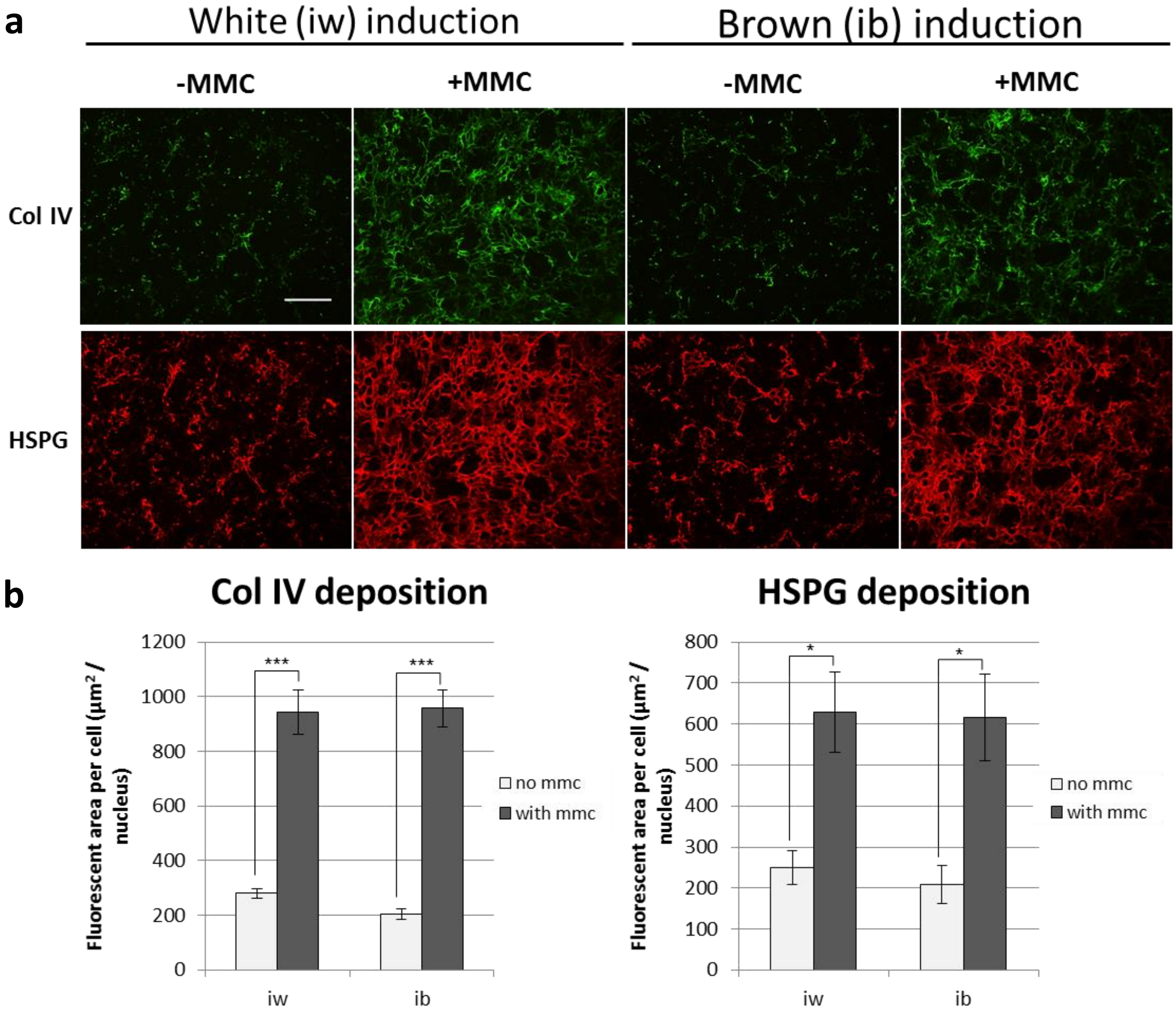

Figure S5. MMC enhances ECM deposition during adipogenic differentiation. bmMSCs were chemically induced with a white (iw) or brown (ib) cocktail ± MMC for 3 weeks and ECM deposition was assessed immunocytochemically. (a) ICC images of deposited collagen IV (Col IV) and heparan sulphate proteoglycan II (perlecan/HSPG). Scale bar: 500µm. (b) Quantitative bioimaging analysis of fluorescent area normalized to cell number. Data are mean ± SEM. *p<0.05; ***p<0.001.

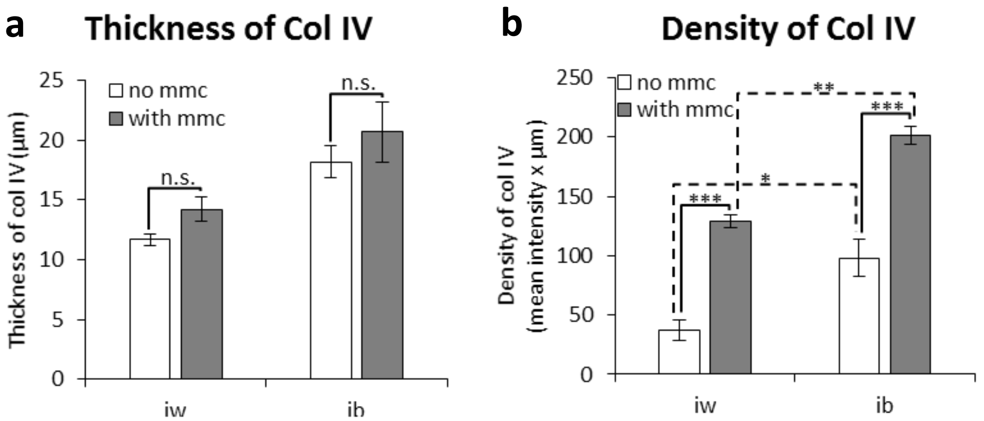

Figure S6. MMC increases the density but not the thickness of Col IV during adipogenic differentiation. bmMSCs were chemically induced with a white (iw) or brown (ib) induction protocol ± MMC for 3 weeks and Z-stack images was obtained for each condition through confocal microscopy. Bio-imaging analysis was performed on 3 Z-stacks per condition to assess the thickness (a) and the density (b) of Col IV deposited. Data are mean ± SEM. n.s. not significant; *p<0.05; **p<0.01; ***p<0.001.

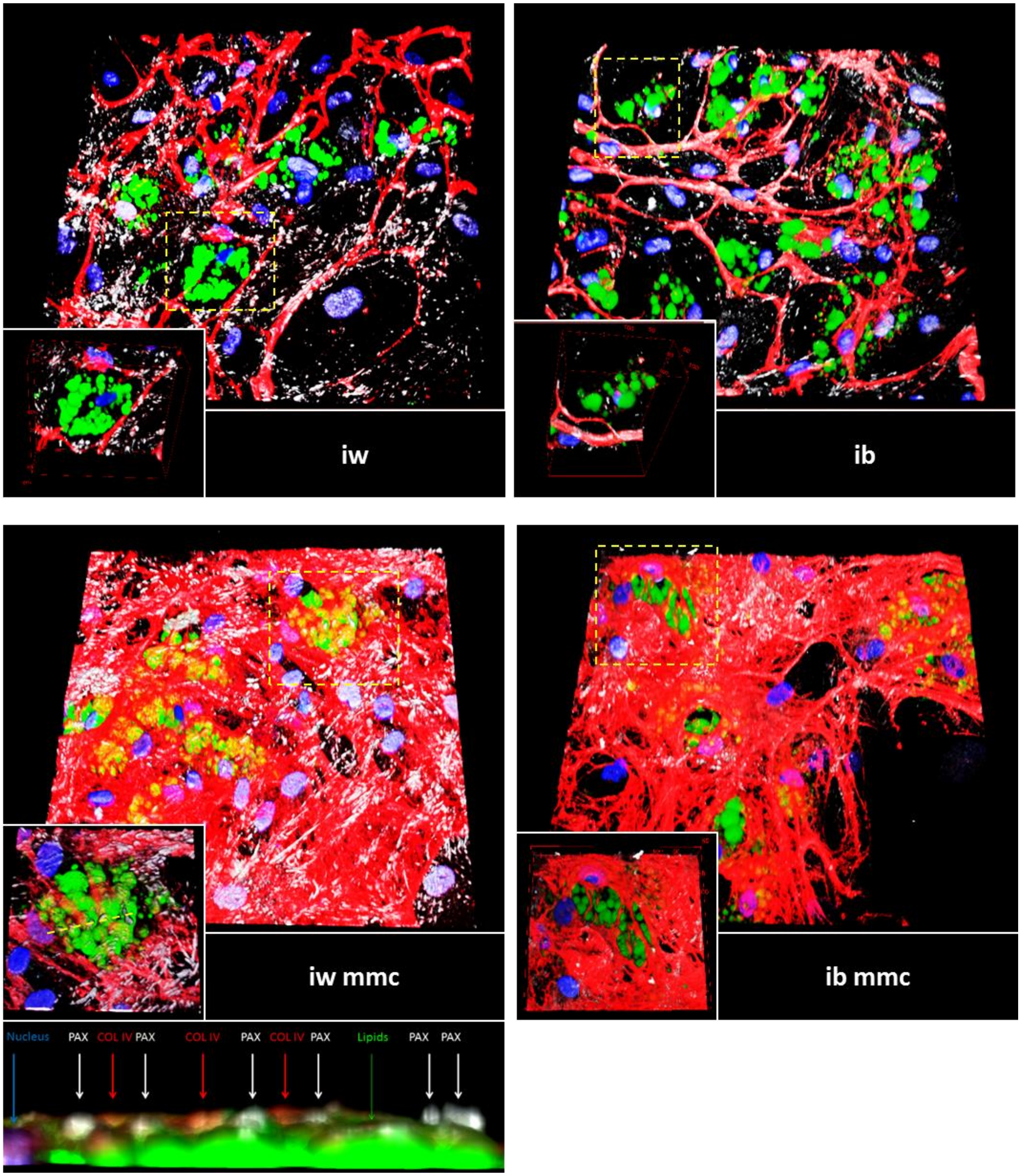

Figure S7. 3D representation of focal adhesion distribution of bmMSC-generated adipocytes. 3D representation of the Z-stack image sets shown in figure 4. Nuclei are depicted in blue, Col IV in red, lipid droplets in green and paxillin in white. Individual slices from the Z-stack are shown in increasing Z-distance from the glass coverslip per condition. Enlarged inserts are taken from the areas boxed in yellow.

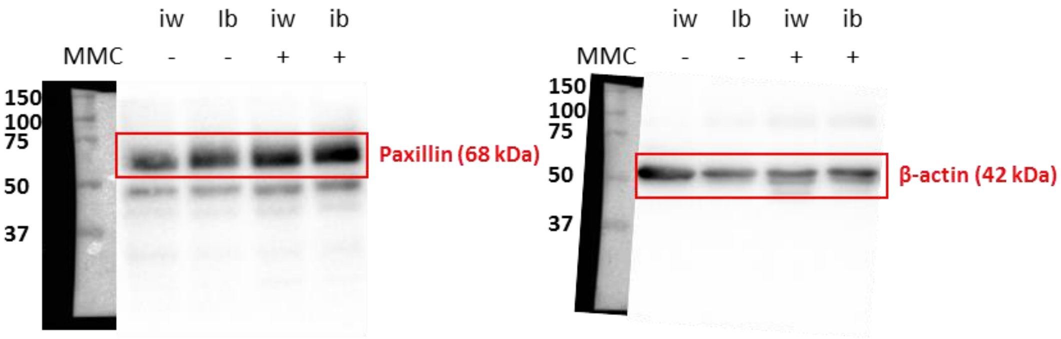

Figure S8. Western blotting of paxillin in bmMSC-derived adipocytes. Western blotting for paxillin was performed on the cell lysates, with β-actin serving as the loading control.

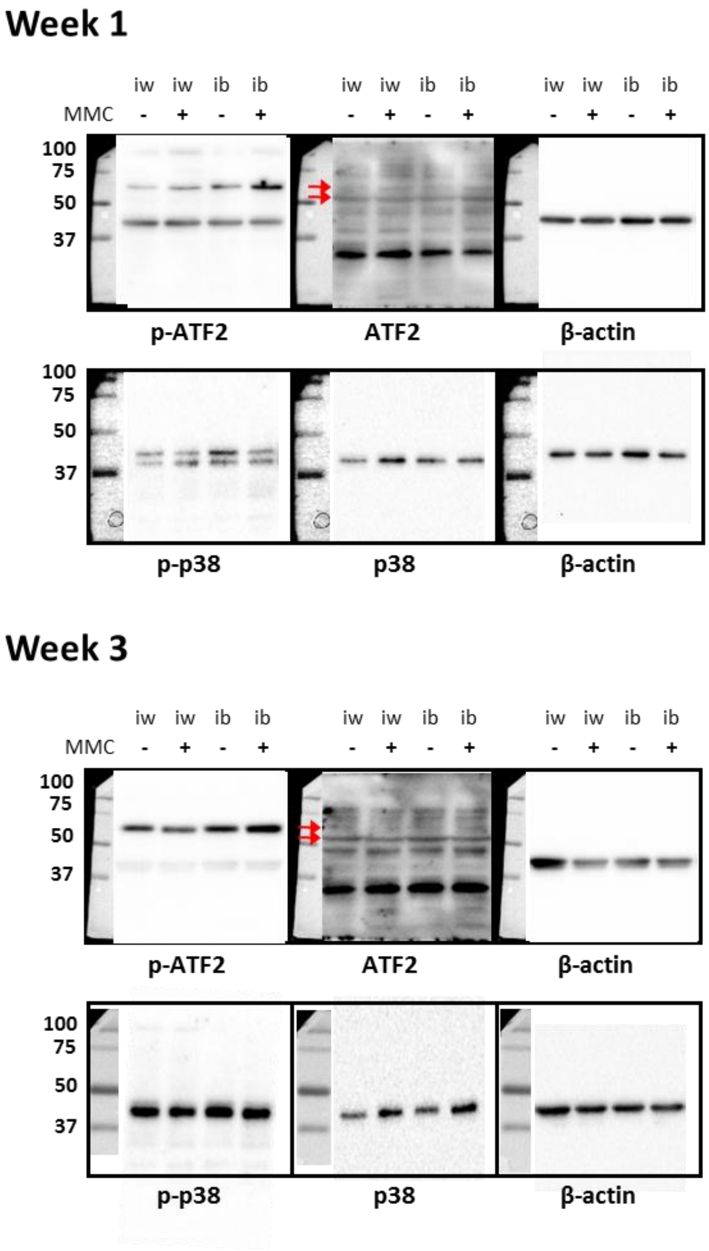

Figure S9. Uncropped p38 and ATF2 Western blots of figure 6. Total ATF2 is identified as the ~55kDa and ~70kDa bands (red arrows).

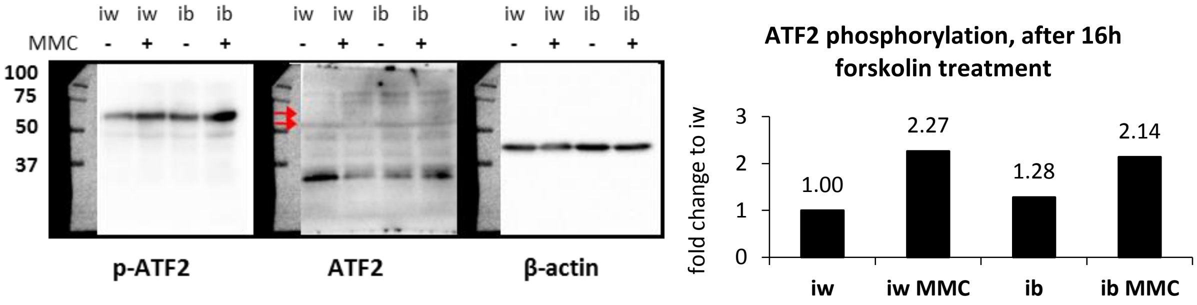

Figure S10. Activation of ATF2 after 16h forskolin stimulation in MMC-generated adipocytes. Western blotting for p-ATF2 and ATF2 were performed on the cell lysates, with β-actin serving as the loading control. Total ATF2 is identified as the ~55kDa and ~70kDa bands (red arrows). Quantification of the ATF2 phosphorylation was performed on these blots and are represented in the bar graphs. N=1.

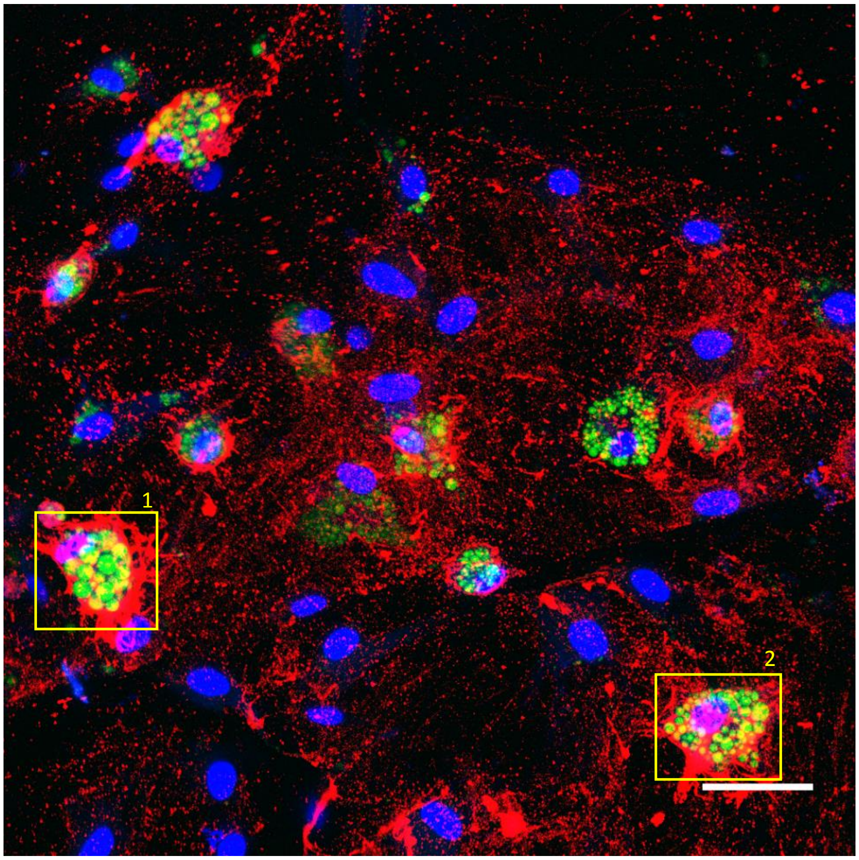

Figure S11. bmMSC-derived adipocytes differentiated in a bovine collagen I hydrogel are encased in self-produced Col IV cocoons. Z-stack images were obtained through confocal microscopy. Nuclei are depicted in blue, Col IV in red and lipid droplets in green. Reconstructed 3D images of the adipocytes boxed in yellow show clearly the encasement of Col IV in the supplementary videos 1 and 2. Scale bar: 50µm.

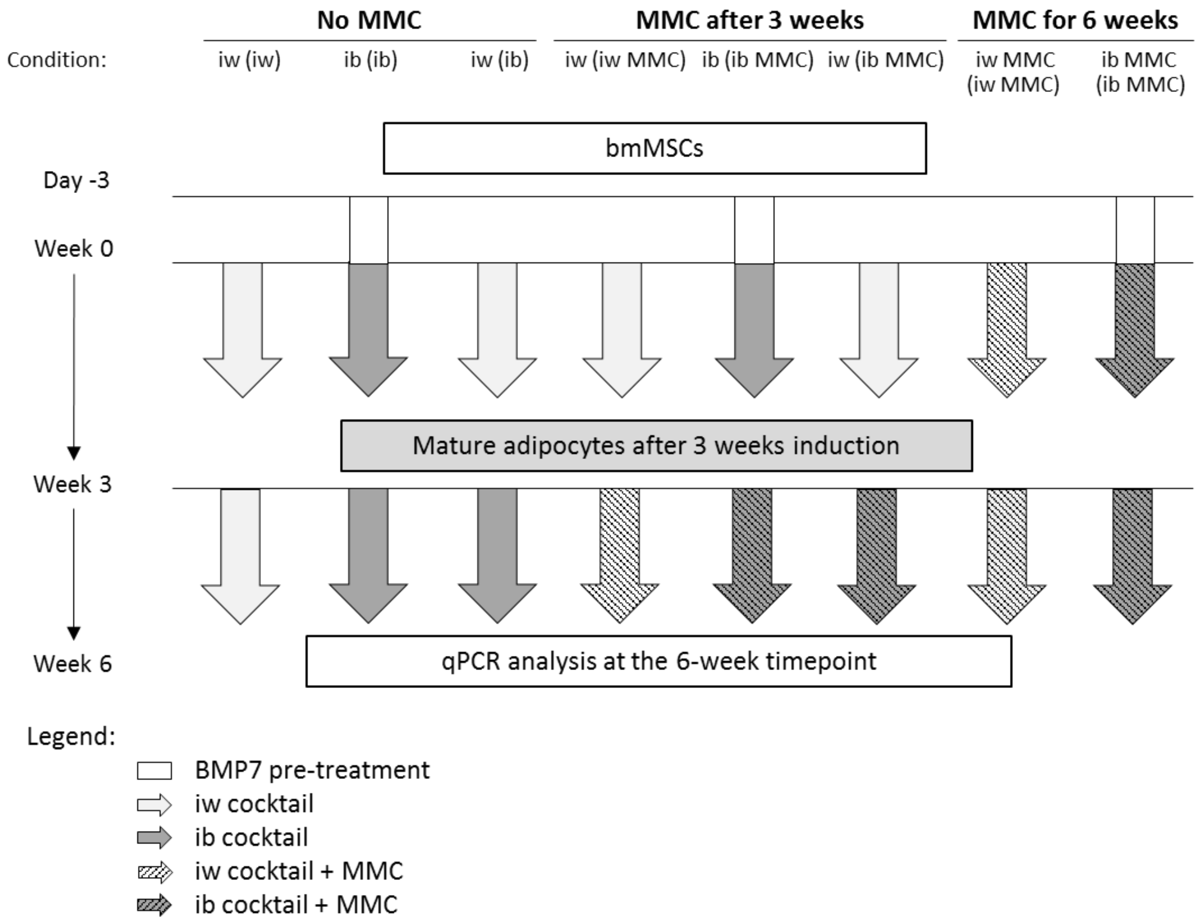

Figure S12. 6-week adipogenic induction protocol to assess browning of bmMSC-derived white adipocytes ±MMC.

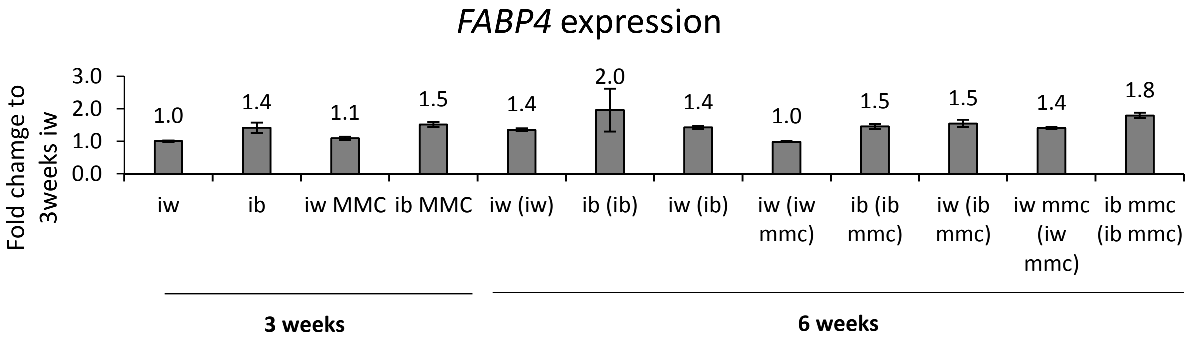

Figure S13a. Comparison of FABP4 mRNA expression in bmMSC-derived adipocytes at 3 and 6 weeks of adipogenic differentiation. qPCR analysis of *FABP4* = fatty acid binding protein 4. Data is expressed as mean ± SEM. There was no significant difference between groups. N = 3.

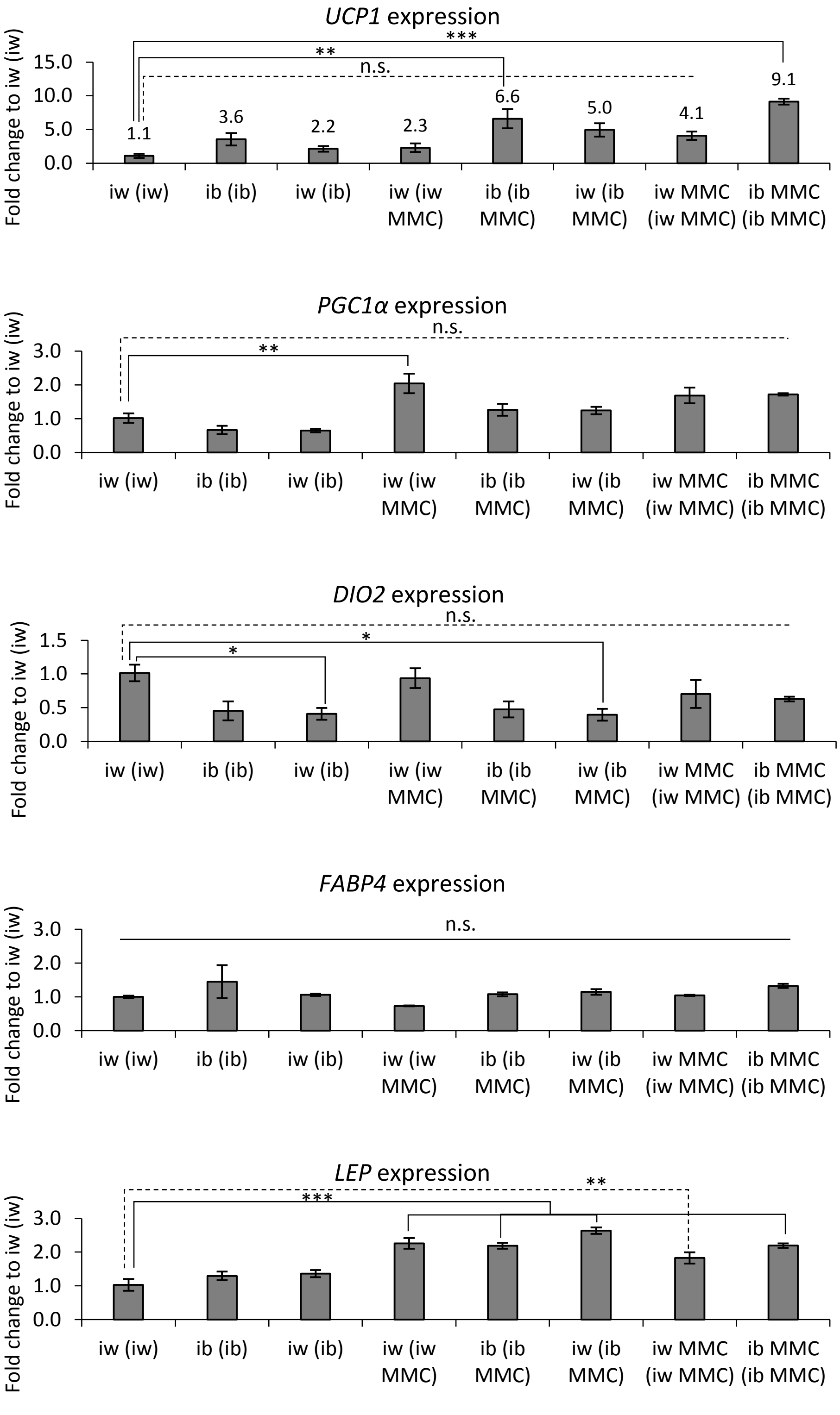

Figure S13b. Browning of WAT-differentiated bmMSCs with MMC as in figure 8. Data is expressed as mean ± SEM. n.s. = not significant; *p<0.05; **p<0.01; ***p<0.001. N = 3, except for the DIO2 iw MMC (iw MMC) condition where N = 2.

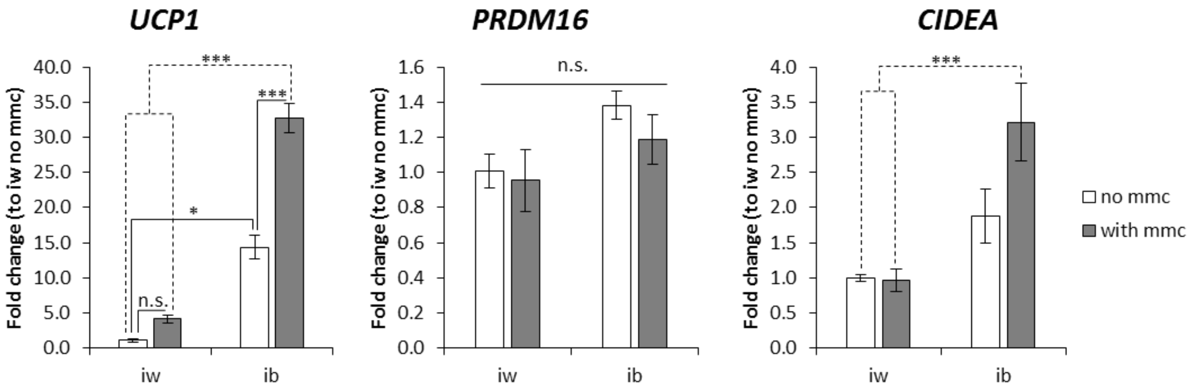

Condition	Ct value
iw	32.2 ± 0.4
ib	29.4 ± 0.1
iw mmc	30.7 ± 0.5
ib mmc	27.4 ± 0.1

Condition	Ct value
iw	27.6 ± 0.2
ib	27.2 ± 0.1
iw mmc	27.6 ± 0.1
ib mmc	27.5 ± 0.1

Condition	Ct value
iw	33.2 ± 0.2
ib	32.4 ± 0.3
iw mmc	33.2 ± 0.1
ib mmc	31.7 ± 0.2

Figure S14. Adipogenic induction under MMC upregulates UCP1 expression in preadipocytes derived from the SVF of adult human subcutaneous abdominal WAT. qPCR analysis of BAT selective genes was carried out: *UCP1* = uncoupling protein 1, *PRDM16* = PRD1-BF1-RIZ1 homologous domain containing 16, *CIDEA* = cell death-inducing DNA fragmentation factor, alpha subunit-like effector a. Data is expressed as mean ± SEM. n.s. = not significant; *$p<0.05$; **$p<0.01$; ***$p<0.001$. N = 3.

a Classical brown

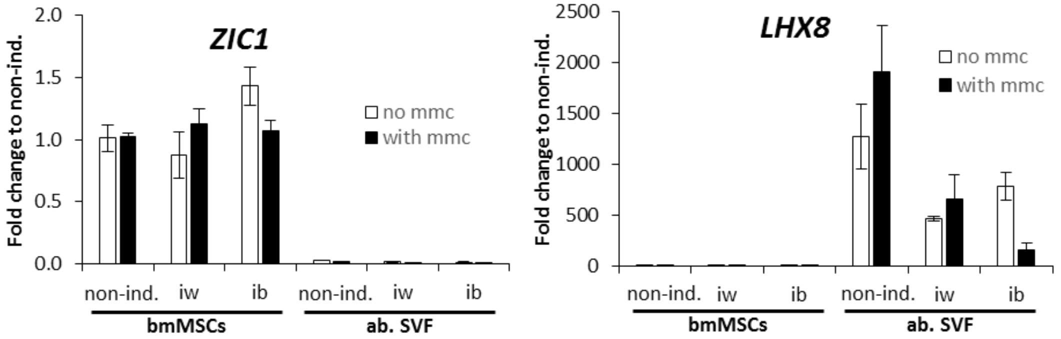

b Brite

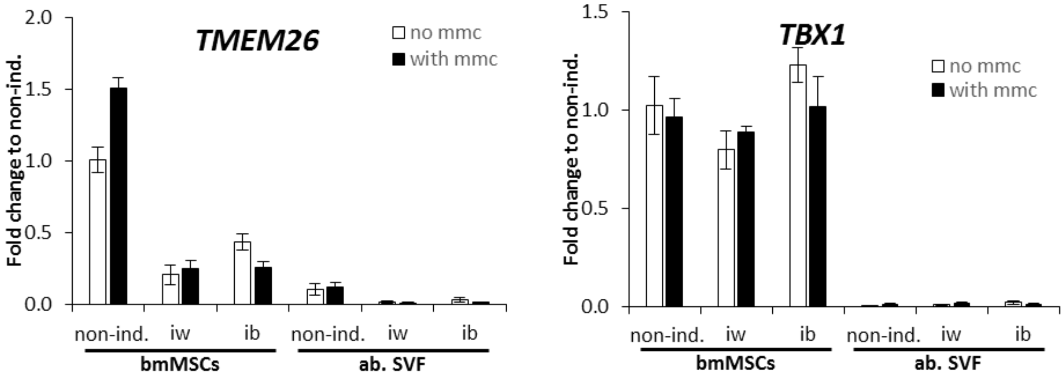

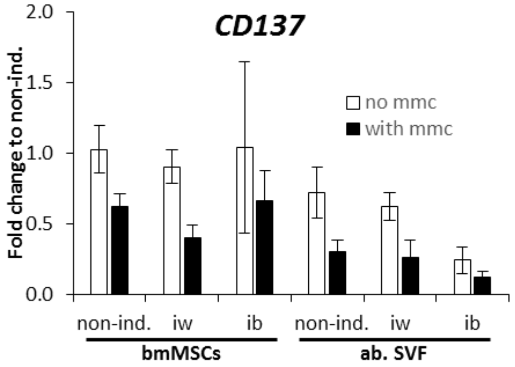

c White

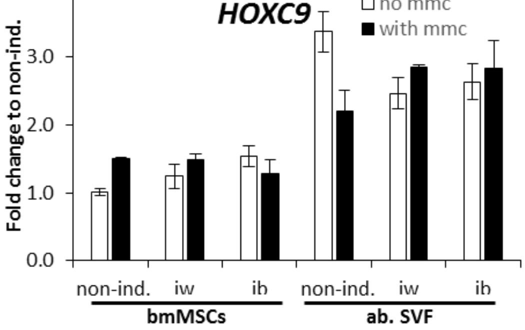

Figure S15. Comparison of classical brown, brite/beige and white markers between bmMSCs and preadipocytes from subcutaneous abdominal WAT. bmMSCs and the SVFs from the subcut. abdominal WAT were chemically induced with a white (iw) or brown (ib) cocktail ±MMC for 3 weeks and qPCR analysis was carried out to compare expression of markers for (a) classical brown adipocytes: *ZIC1* = Zic family member 1, LHX8 = *LIM homeobox protein 8*; (b) brite / beige adipocytes: *TMEM26* = transmembrane protein 26, *TBX1* = T-box 1, *CD137* = tumor necrosis factor receptor superfamily, member 9 (TNFRSF9); and (c) white adipocytes: *HOXC9* = homeobox C9. Data is expressed as mean ± SEM. N = 3. Note that for LHX8, the ib MMC SVF condition was done in duplicate.

Table S1. Ct values of FGF21 in bmMSC-derived adipocytes after a 4h forskolin stimulation.

FGF21	iw	ib	iw MMC	ib MMC	iw f	ib f	iw MMC f	ib MMC f
1	35 (no Ct)	35 (no Ct)	31.86	35 (no Ct)	32.59	35 (no Ct)	34.17	32.51
2	35 (no Ct)	35 (no Ct)	34.57	35 (no Ct)	35 (no Ct)	35 (no Ct)	32.6	32.75
3	35 (no Ct)	35 (no Ct)	35 (no Ct)	35 (no Ct)	35 (no Ct)	-	30.99	30.46

Table S2. Ct values of classical brown, brite/beige and white markers in bmMSCs and preadipocytes from subcutaneous abdominal WAT. Raw Ct values of the various genes reported in figure S12.

ZIC1	bmMSCs						ab. SVFs					
	**ctrl**	**iw**	**ib**	**ctrl mmc**	**iw mmc**	**ib mmc**	**ctrl**	**iw**	**ib**	**ctrl mmc**	**iw mmc**	**ib mmc**
1	24.09	24.78	24.49	24.32	24.40	24.47	29.71	30.25	30.59	30.19	31.28	31.24
2	24.66	24.46	24.81	24.40	24.13	24.47	29.41	29.89	30.28	30.00	30.52	31.10
3	24.73	24.75	24.52	24.31	24.27	25.02	29.43	30.76	31.07	29.97	31.04	30.99
**Average**	**24.49**	**24.66**	**24.61**	**24.34**	**24.27**	**24.65**	**29.52**	**30.30**	**30.65**	**30.05**	**30.95**	**31.11**
SE	0.20	0.10	0.10	0.03	0.08	0.18	0.10	0.25	0.23	0.07	0.22	0.07
LHX8	**bmMSCs**						**ab. SVFs**					
	**ctrl**	**iw**	**ib**	**ctrl mmc**	**iw mmc**	**ib mmc**	**ctrl**	**iw**	**ib**	**ctrl mmc**	**iw mmc**	**ib mmc**
1	35	35	35	35	35	35	25.67	26.23	25.46	24.78	26.73	28.23
2	35	35	35	35	31.55	30.84	24.04	25.71	25.8	23.13	24.86	27.41
3	35	35	35	32.03	33.17	35	24.67	26.32	25.46	23.65	25.68	-
**Average**	**35.00**	**35.00**	**35.00**	**34.01**	**33.24**	**33.61**	**24.79**	**26.09**	**25.57**	**23.85**	**25.76**	**27.82**
SE	0.00	0.00	0.00	0.99	1.00	1.39	0.47	0.19	0.11	0.49	0.54	0.41
TMEM26	**bmMSCs**						**ab. SVFs**					
	**ctrl**	**iw**	**ib**	**ctrl mmc**	**iw mmc**	**ib mmc**	**ctrl**	**iw**	**ib**	**ctrl mmc**	**iw mmc**	**ib mmc**
1	27.43	30.88	29.62	26.95	29.97	29.77	30.36	35	33.02	30.2	34.01	34.22
2	27.96	29.88	29.74	27.27	29.99	30.1	31.45	33.72	35	31.31	35	33.57
3	27.96	29.82	29.51	27.01	29.36	30.23	31.76	32.88	32.19	30.51	33.7	34.46
**Average**	**27.78**	**30.19**	**29.62**	**27.08**	**29.77**	**30.03**	**31.19**	**33.87**	**33.40**	**30.67**	**34.24**	**34.08**
SE	0.18	0.34	0.07	0.10	0.21	0.14	0.42	0.62	0.83	0.33	0.39	0.27
TBX1	**bmMSCs**						**ab. SVFs**					
	**ctrl**	**iw**	**ib**	**ctrl mmc**	**iw mmc**	**ib mmc**	**ctrl**	**iw**	**ib**	**ctrl mmc**	**iw mmc**	**ib mmc**
1	26.91	27.4	27.49	27.2	27.37	27.33	35	33.81	32.41	33.78	35	32.5
2	27.45	27.69	27.88	27.27	27.47	27.69	35	33.8	32.87	33.49	33.09	35
3	27.75	27.77	27.71	27.48	27.59	27.87	35	35	35	32.81	32.43	35
**Average**	**27.37**	**27.62**	**27.69**	**27.32**	**27.48**	**27.63**	**35.00**	**34.20**	**33.43**	**33.36**	**33.51**	**34.17**
SE	0.25	0.11	0.11	0.08	0.06	0.16	0.00	0.40	0.80	0.29	0.77	0.83
CD137	**bmMSCs**						**ab. SVFs**					
	**ctrl**	**iw**	**ib**	**ctrl mmc**	**iw mmc**	**ib mmc**	**ctrl**	**iw**	**ib**	**ctrl mmc**	**iw mmc**	**ib mmc**
1	30.39	30.7	33.69	31.2	32.01	31.8	31.12	31.64	32.53	32.2	32.89	34.26
2	31.32	31.39	32.25	31.77	31.75	32.49	31.3	31.88	33.67	33.05	32.06	35
3	31.23	31.06	30.63	31.73	33.15	31.69	32.24	31.44	33.83	32.28	35	33.6
**Average**	**30.98**	**31.05**	**32.19**	**31.57**	**32.30**	**31.99**	**31.55**	**31.65**	**33.34**	**32.51**	**33.32**	**34.29**
SE	0.30	0.20	0.88	0.18	0.43	0.25	0.35	0.13	0.41	0.27	0.88	0.40
HOXC9	**bmMSCs**						**ab. SVFs**					
	**ctrl**	**iw**	**ib**	**ctrl mmc**	**iw mmc**	**ib mmc**	**ctrl**	**iw**	**ib**	**ctrl mmc**	**iw mmc**	**ib mmc**
1	24.18	23.85	24.26	23.69	23.68	24.21	22.6	22.83	23.14	22.85	22.85	23.15
2	24.44	23.94	24.49	23.79	23.66	24.28	22.74	23.01	23.07	22.9	22.87	22.79
3	24.44	24.14	24.32	23.47	23.79	24.39	22.5	23.2	23.1	22.95	22.6	23.13
**Average**	**24.35**	**23.98**	**24.36**	**23.65**	**23.71**	**24.29**	**22.61**	**23.01**	**23.10**	**22.90**	**22.77**	**23.02**
SE	0.09	0.09	0.07	0.09	0.04	0.05	0.07	0.11	0.02	0.03	0.09	0.12

Table S3. List of primers used in qPCR analysis.

Gene	Accession no.	Reference	Forward primer	Reverse Primer
RPLP0	NM_001002.3	1	CACCATTGAAATCCTGAGTGATGT	TGACCAGCCCAAAGGAGAAG
TBP	NM_003194.4	2	CACGAACCACGGCACTGATT	TTTTCTTGCTGCCAGTCTGGAC
FABP4	NM_001442.2		TGTGCAGAAATGGGATGGAAA	CAACGTCCCTTGGCTTATGCT
GLUT4	NM_001042.2	3	TCAACAATGTCCTGGCGGTG	TTCTGGATGATGTAGAGGTAGCGG
HSL	NM_005357.2	4	CTCAGTGTGCTCTCCAAGTG	CACCCAGGCGGAAGTCTC
LEP	NM_000230.2	5	TTTGGCCCTATCTTTTCTATGTCC	TGGAGGAGACTGACTGCGTG
UCP1	NM_021833.4	6	CTGGAATAGCGGCGTGCTT	AATAACACTGGACGTCGGGC
PRDM16	NM_022114.3		GAGGAGGACGATGAGGACAG	CGGCTCCAAAGCTAACAGAC
PGC1α	NM_013261.3		GCCAAACCAACAACTTTATCTCTTC	CACACTTAAGGTGCGTTCAATAGTC
DIO2	NM_013989.4		CCTCCTCGATGCCTACAAAC	GCTGGCAAAGTCAAGAAGGT
CIDEA	NM_001279.3	7	GGCAGGTTCACGTGTGGATA	GAAACACAGTGTTTGGCTCAAGA
TBX1	NM_080646.1	8	ACGACAACGGCCACATTATTC	CCTCGGCATATTTCTCGCTATCT
CD137	NM_001561.5		AGCTGTTACAACATAGTAGCCAC	TCCTGCAATGATCTTGTCCTCT
TMEM26	NM_178505.6		ATGGAGGGACTGGTCTTCCTT	CTTCACCTCGGTCACTCGC
ZIC1	NM_003412.3	9	GCATCCCAGTTCGCTGCGCAAA	GGAGACACGATGGTGGGAGGCG
HOXC9	NM_006897.1		GCAGCAAGCACAAAGAGGAGAAG	GCGTCTGGTACTTGGTGTAGGG
LHX8	NM_001001933.1	10	ACAACCCAGATGCACAGACA	TGTGGCGTGCTCTACAATTC
FGF21	NM_019113.2	11	ACTCCAGTCCTCTCCTGCAA	GCACAGGAACCTGGATGTCT

Supplementary Videos

Supplementary video 1 – 3D reconstructed image of a bmMSC-derived adipocyte differentiated in a collagen I gel indicated in box 1 of supplementary figure S11. Nuclei are depicted in blue, Col IV in red and lipid droplets in green.

Supplementary video 2 – 3D reconstructed image of a bmMSC-derived adipocyte differentiated in a collagen I gel indicated in box 2 of supplementary figure S11. Nuclei are depicted in blue, Col IV in red and lipid droplets in green.

Supplementary Experimental Procedures

Quantitation of collagen IV from the Z-stack images – Images of the collagen IV staining were processed using the Image J software (National Institutes of Health, Bethesda, MD, USA) to remove background noise and calculate the average pixel intensity per confocal plane, which was then plotted against the Z-distance (µm) for each image stack. Assuming a normal distribution, the parabolic section of the resulting curve was then fitted to a Gaussian function (equation 1) using the area under the curve (A), the peak position (µ) and the standard deviation (σ) as floating parameters. Following the density of probability model, the limit of the collagen IV signal along the Z-axis was determined as (µ+3σ), with a 99% confidence level, and the corresponding Z-distance was used to estimate the thickness of collagen IV. The area under the curve up to the limit was obtained using OriginPro 9.1 (OriginLab Corporation, Northhampton, MA, USA) to estimate the density of collagen IV (figure below).

$$y = \frac{A}{\sigma\sqrt{2\pi}} e^{-0.5\times(\frac{x-\mu}{\sigma})^2} \qquad \text{(eq.1)}$$

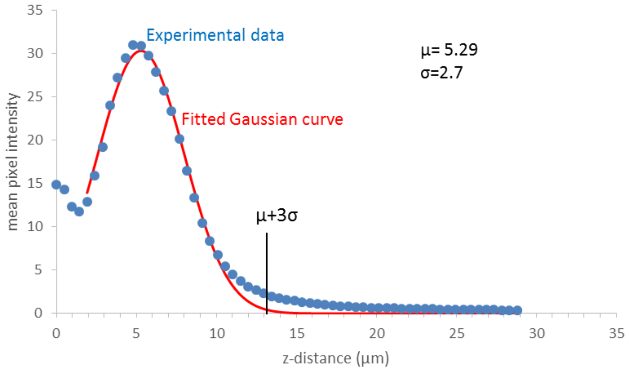

Supplementary References

1 Jansen, P. A. *et al.* Expression of the vanin gene family in normal and inflamed human skin: induction by proinflammatory cytokines. *J. Invest. Dermatol.* **129**, 2167-2174, (2009).

2 Elabd, C. *et al.* Oxytocin controls differentiation of human mesenchymal stem cells and reverses osteoporosis. *Stem Cells* **26**, 2399-2407, (2008).

3 Lee, E. K. *et al.* miR-130 suppresses adipogenesis by inhibiting peroxisome proliferator-activated receptor gamma expression. *Mol. Cell. Biol.* **31**, 626-638, (2011).

4 Mairal, A., Langin, D., Arner, P. & Hoffstedt, J. Human adipose triglyceride lipase (PNPLA2) is not regulated by obesity and exhibits low in vitro triglyceride hydrolase activity. *Diabetologia* **49**, 1629-1636, (2006).

5 Degawa-Yamauchi, M. *et al.* Regulation of adiponectin expression in human adipocytes: effects of adiposity, glucocorticoids, and tumor necrosis factor alpha. *Obes. Res.* **13**, 662-669, (2005).

6 Virtanen, K. A. *et al.* Functional brown adipose tissue in healthy adults. *N. Engl. J. Med.* **360**, 1518-1525, (2009).

7 Sharp, L. Z. *et al.* Human BAT possesses molecular signatures that resemble beige/brite cells. *PLoS One* **7**, e49452, (2012).

8 Wu, J. *et al.* Beige adipocytes are a distinct type of thermogenic fat cell in mouse and human. *Cell* **150**, 366-376, (2012).

9 Lidell, M. E. *et al.* Evidence for two types of brown adipose tissue in humans. *Nat. Med.* **19**, 631-634, (2013).

10 Cypess, A. M. *et al.* Anatomical localization, gene expression profiling and functional characterization of adult human neck brown fat. *Nat. Med.* **19**, 635-639, (2013).

11 Dushay, J. *et al.* Increased fibroblast growth factor 21 in obesity and nonalcoholic fatty liver disease. *Gastroenterology* **139**, 456-463, (2010).
